# Menopause Analytical Hormonal Correlate Outcome Study (MAHCOS) and the Association to Brain Electrophysiology (P300) in a Clinical Setting

**DOI:** 10.1371/journal.pone.0105048

**Published:** 2014-09-24

**Authors:** Eric R. Braverman, David Han, Marlene Oscar-Berman, Tatiana Karikh, Courtney Truesdell, Kristina Dushaj, Florian Kreuk, Mona Li, Danielle Stratton, Kenneth Blum

**Affiliations:** 1 Department of Clinical Neurology, PATH Foundation NY, New York, New York, United States of America; 2 Department of Psychiatry, University of Florida, College of Medicine and McKnight Brain Institute, Gainesville, Florida, United States of America; 3 Department of Management Science and Statistics, University of Texas at San Antonio, San Antonio, Texas, United States of America; 4 Departments of Psychiatry, Neurology, and Anatomy & Neurobiology, Boston University School of Medicine, and Boston VA Healthcare System, Boston, Massachusetts, United States of America; 5 Department of Psychiatry, Human Integrated Services Unit, University of Vermont, Center for Clinical and Translational Science, Burlington, Vermont, United States of America; 6 Institute of Integrative Omics and Applied Biotechnology, Nonakuri, Purba Medinipur, West Bengal, India; 7 Dominion Diagnostics, LLC., North Kingstown, Rhode Island, United States of America; University of Manchester, United Kingdom

## Abstract

Various studies have demonstrated that increased leptin levels and obesity are inversely related to cognitive decline in menopausal women. It is hypothesized that adiposity is inversely correlated with cognitive decline, as women with increased weight are less vulnerable to diminishing cognition. However, it is increasingly observed that menopausal women, even with increased adiposity, experience significant cognitive decline. Positron emission tomography (PET) has been used to analyze cognitive function and processing in menopausal women. Evoked potentials (P300) and neurophysiologic tests have validated brain metabolism in cognitively impaired patients. Post-hoc analyses of 796 female patients entering PATH Medical Clinic, between January 4, 2009 and February 24, 2013, were performed as part of the “Menopause Analytical Hormonal Correlate Outcome Study” (MAHCOS). Patient age range was 39–76 years (46.7±0.2). P300 latency and amplitude correlated with a number of hormones: follicle stimulating hormone (FSH), luteinizing hormone (LH), estradiol, estrone, estriol, DHEA, pregnenolone, progesterone, free and total testosterone, thyroid stimulating hormone (TSH), Vitamins D 1.25 and D 25OH, leptin, and insulin-like growth factor-binding protein 3 (IGF-BP3). Corrected statistics did not reveal significant associations with P300 latency or amplitude for these hormones except for leptin plasma levels. However, factor analysis showed that FSH and LH clustered together with Vitamin D1.25 and Vitamin D25OH, P300 latency (not amplitude), and log leptin were found to be associated in the same cluster. Utilizing regression analysis, once age adjusted, leptin was the only significant predictor for latency or speed (p = 0.03) with an effect size of 0.23. Higher plasma leptin levels were associated with abnormal P300 speed (OR = 0.98). Our findings show a significant relationship of higher plasma leptin levels, potentially due to leptin resistance, and prolonged P300 latency. This suggests leptin resistance may delay electrophysiological processing of memory and attention, which appears to be the first of such an association.

## Introduction

Menopausal women become deficient in multiple hormones such as estrogen, progesterone, testosterone, and dehydroepiandrosterone (DHEA) [1]–[6] with increases in luteinizing hormone (LH), follicle stimulating hormone (FSH), and thyroid stimulating hormone (TSH) [7]–[10]. All of these hormones have individual as well as inter-related functions in the human body, including pulmonary, cardiovascular, gastrointestinal, and immunological functions [11], [12]. Aging is continually changing as life expectancy increases, and the decrease in cognition through normal aging processes is of primary clinical interest [6], [13]. In fact, estrogen deficiency has been proposed as a cause of memory deficits in postmenopausal women [14]. There are studies that suggest that LH increases after menopause with concomitant decline in cognitive performance [15]. Chorionic gonadotropin receptors and LH occur in the brain [16]. Thus, levels of LH and FSH may increase low-density lipoprotein receptor-related protein in the brain [14], [17]. Levels of FSH increase dramatically in women during and after menopause and can be lowered with estrogen therapy [14], [18]. Emerging evidence suggests that high TSH levels are associated with a two-fold risk of cognitive decline as well as prevalence of anomalies in musculoskeletal systems [19]–[21].

### Menopause and Leptin

Weight gain, and associated leptin level changes during menopause have been demonstrated in numerous cases, as leptin was found to have significant association with metabolic factors when compared to resistin in pre and post-menopausal women [24]–[33]. In particular menopausal status is linked with increased weight gain, increased central fat mass, and abnormal lipid metabolism. One case observed a significant statistical correlation with serum leptin levels and fat mass; abdominal obesity and visceral fat area were positively correlated with serum leptin level [28]. Leptin concentration was positively correlated with BMI and weight gain in healthy menopausal women [86]. There is indeed a definitive correlation with plasma leptin levels in peri- and postmenopausal women and weight gain, which suggests that there is a dynamic interaction between plasma leptin and body composition. These changes may further indicate that a significant association with leptin levels and metabolic factors could impact other hormonal composition changes that occur during menopause.

One important question that concerns menopausal women is weight gain and early cognitive decline [22]. In some studies, researchers have found that obesity-related waist circumference had a positive correlation with global cognition [23]. There have been controversial reports indicating that naturally occurring estrogen may have a beneficial effect on cognition in women [33]–[37]. There is a strong relationship between the hormone leptin and obesity especially in females [36]–[39]; however, leptin's role in cognition has been studied with mixed results [28]–[30] and there is a notable lack of published data on P300 utilization and its relationship with plasma leptin levels, despite studies indicating the association between leptin and cognitive genes. The P300 evoked potential measures electrical brain waves and their function in particular regions of the brain. It is a diagnostic tool used to identify neurological deficits or abnormalities, such as cognitive decline and neurotransmitter deficiencies.

Moreover delayed P300 latency and obesity are both associated with dementia. Leptin resistance may delay electrophysiological processing of memory and attention. [87].

### The Clinical Significance of P300 Testing

When used in a clinical setting, the P300 serves as an invaluable resource towards early diagnosis and treatment of poor cognition and loss of brain vitality due to menopausal transition. Analysis of the P300 and standard neuropsychological variables to assess patients with mild cognitive impairment (MCI) and Alzheimer's disease can provide valuable information for the detection of MCI and AD [80]. A number of studies determine that there is justification and efficacy in employing more resources unto converting P300 as an acceptable biological marker for AD [81]–[84]. P300 is responsive to the deterioration of language, memory, and executive functions, thereby acting as a useful tool in evaluating cognitive function [85]. The P300 was therefore a suitable tool in evaluating the variations in cognitive function and quality in subjects.

We hypothesized that women presenting with complaints related to menopause would have a number of associated hormonal changes relating specifically to both somatic and neurological symptoms. We also hypothesized that there would be an inverse relationship between plasma leptin levels and P300 speed and voltage based on literature suggesting that as women transition into menopause, they are more likely to gain weight due to associated changes leptin levels, which have been associated with a decrease in cognitive functioning [40]–[43]. In this paper, we focus on the role of leptin plasma levels and brain electrophysiology as measured by P300 amplitude (voltage) and latency.

## Methods

### Subjects

All female patients who presented to a private clinic, at or approaching the typical age of menopause onset (age 39–59), were examined. We also extended this study to include a small number of women (n = 33) between 35 to 39 years, as well as 10 women ≥60 years. The descriptive distribution summary of each measured variable collected from January 4, 2009 to February 24, 2013 in this retrospective study is as follows: The initial sample size was n = 796 female patients. Missing values are common in this dataset, and certain variables were dichotomized based on the cutoff chart with the normal ranges given. Each patient filled out an approved PATH Foundation NY IRB (PFNYIRB) informed consent form, and the PFNYIRB committee approved the study [Registration # IRB00002334; Protocol #: LEXMEN001].

**Figure 1:**
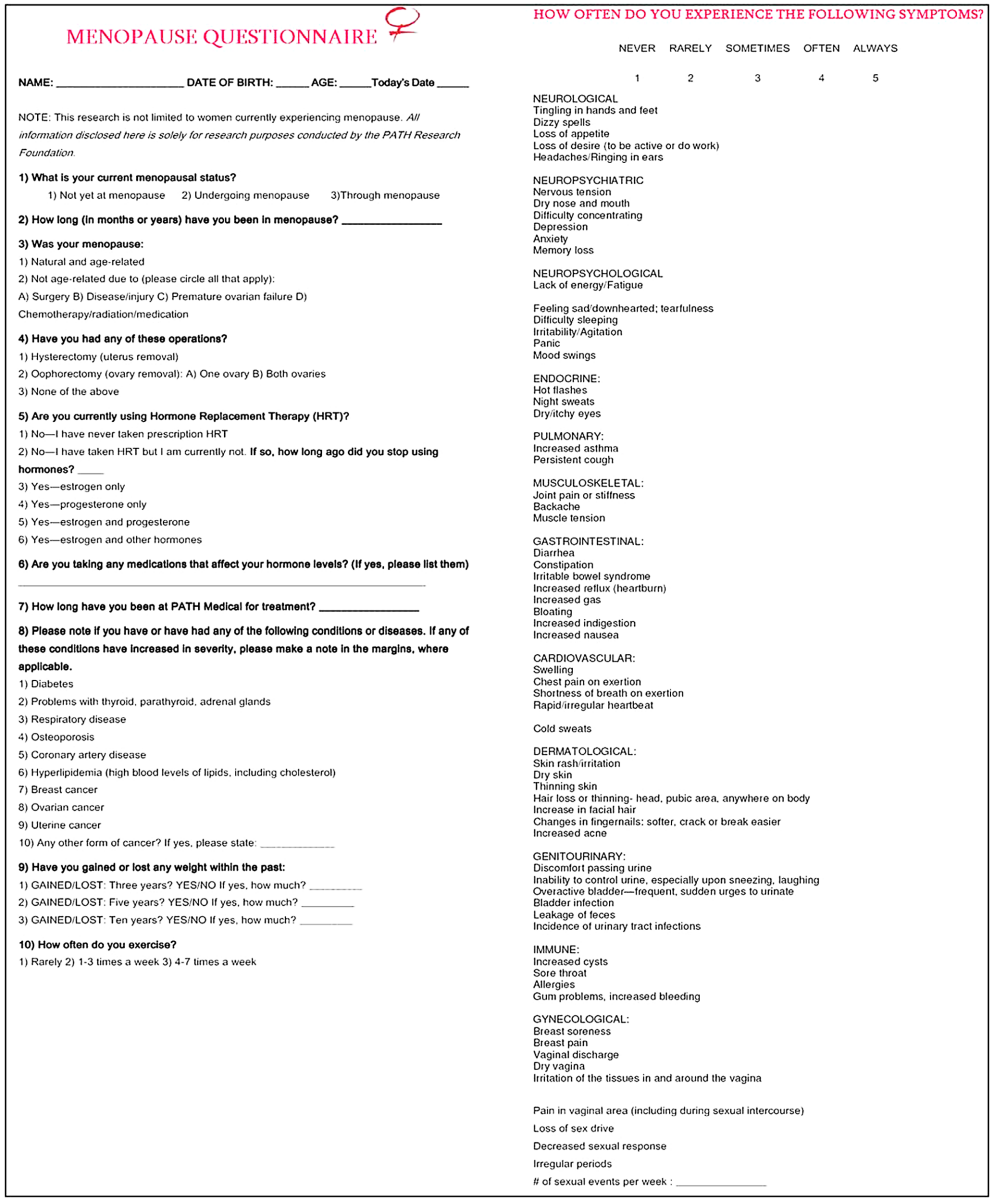
Menopause Questionnaire.

All subjects underwent a thorough medical evaluation including a full screen for hormones including DHEA sulfate, estradiol, estrone, FSH, LH, pregnenolone, progesterone, free and total testosterone, and TSH obtained from BioReference Laboratories, Inc. Hormone blood levels were drawn between 9:30 am and 3:00 pm. A detailed medical history was obtained including information on the stage of menopause (pre, undergoing, or post), origin of menopause, and history of hormone replacement therapy as well as anthropometric characteristics such as weight, height, BMI, body fat percentage, muscle, and bone density recorded via duel-energy x-ray absorptiometry (DXA). These anthropometric characteristics were not included in the study's final data analysis, but may be considered for future studies. A Menopause Questionnaire (Figure 1) was given to all women (n = 796) following a preliminary screening. The quantitative section of the Menopause Questionnaire consisted of 64 questions related to symptoms of menopause. Each symptom was rated on a Likert scale of frequency from 1 (never) to 5 (always). The total number of endorsed symptoms was calculated as a gross indicator of menopausal symptomatology. Mean values of the Likert ratings also were calculated within each of 12 domains of symptoms: neurological, neuropsychiatric, neuropsychological, endocrine, pulmonary, musculoskeletal, gastrointestinal, cardiovascular, immune, dermatological, genitourinary, and gynecological. A grand mean of the Likert rating across all domains also was calculated. All reported blood levels and preliminary questionnaires were recorded within 3 weeks of each other.

The demographics for the study are outlined in Table 1 (subsections A–S). The age range of the patients was 39–76 years (46.7±0.2 years, n = 796) with ∼93.6% of patients between ages 40 and 54 (n = 745); FSH levels were 30.1±1.3 mIU/mL (n = 731) with strong right skewed distribution; LH levels were 19.1±0.7 mIU/mL (n = 726) with strong right skewed distribution; TSH levels were 2.0±0.2 µIU/mL (n = 795) with strong right distribution; leptin levels were 21.0±0.8 ng/mL (n = 565) with strong right skewed distribution; IGF-BP3 levels were 4.4±0.1 µg/mL (n = 306) with moderately normal distribution; Vitamin D 25OH levels were 37.5±0.8 ng/mL (n = 671) with moderately right skewed distribution; Vitamin D 1,25 levels were 61.4±1.1 pg/mL (n = 585) with moderately right skewed distribution; % Free Testosterone, Testosterone Free, and Testosterone Total levels were 2.9±0.4% (n = 26), 0.8±0.1 pg/mL (n = 711), and 30.8±1.5 ng/dL (n = 715), respectively, Testosterone Free and Testosterone Total with strong right skewed distributions; estradiol levels were 97.4±7.4 pg/mL (n = 740) with strong right skewed distribution; estrone levels were 69.7±4.2 pg/mL (n = 522) with strong right skewed distribution; estriol levels were 0.2±0.0 ng/mL (n = 513); ∼24% of patients underwent Brain Electrical Activity Mapping (BEAM) (n = 796); P300 latency levels were 319.7±1.9 ms (n = 194) with moderately normal distribution; and, P300 amplitude levels were 4.8±0.4 µv (n = 194) with strong right skewed distribution.

**Table 1:** A–S: Demographics of Subjects.

**A: Age Demographics of Subjects With Age Range of Patients 39–76 years (46.7±0.2 years) (n = 796)**		
Age Group	Count	Proportion
2: 35–39	33	0.04146
3: 40–44	217	0.27261
4: 45–49	321	0.40327
5: 50–54	207	0.26005
6: 55–59	8	0.01005
7: 60–64	8	0.01005
A 9: 71+	2	0.00251
Total	796	1
**B: Dichotomized FSH Level Demographics of Subjects with 30.1±1.3 (n = 731) strongly right skewed distribution**		
FSH Level Dichotomized	Count	Proportion
Pre-menopausal	428	0.5855
Post-menopausal	303	0.4145
Total	731	1
**C: Demographics for Subjects with FSH Values Below and Over 40**		
FSH Value over 40?	Count	Proportion
Yes	212	0.29001
No	519	0.70999
Total	731	1
**D: Demographics for Subjects with FSH Values Below and Over 80**		
FSH Value over 80?	Count	Proportion
Yes	92	0.12585
No	639	0.87415
Total	731	1
**E: Demographics for Subjects with FSH Values Below and Over 100**		
FSH Value over 100?	Count	Proportion
Yes	48	0.06566
No	683	0.93434
Total	731	1
**F: Dichotomized LH Level Demographics for Subjects (19.1±0.7; n = 726; strongly right skewed distribution)**		
LH Level Dichotomized	Count	Proportion
Pre-menopausal	378	0.52066
Post-menopausal	348	0.47934
Total	726	1
**G: Dichotomized TSH Level Demographics for Subjects (2.0±0.2; n = 795; strongly right skewed distribution)**		
TSH Level Dichotomized	Count	Proportion
Normal	728	0.91572
Abnormal	67	0.08428
Total	795	1
**H: Dichotomized Leptin Level Demographics for Subjects (21.0±0.8; n = 565; strongly right skewed distribution)**		
Leptin Level Dichotomized	Count	Proportion
Normal	260	0.46018
Abnormal	305	0.53982
Total	565	1
**I: Dichotomized IGF-BP3 Level Demographics for Subjects (4.4±0.1; n = 306; moderately normal distribution)**		
IGF-BP3 Level Dichotomized	Count	Proportion
Normal	287	0.93791
Abnormal	19	0.06209
Total	306	1
**J: Dichotomized Vitamin D 250H Level Demographics for Subjects (37.5±0.8; n = 671; moderately right skewed distribution)**		
Vitamin D 25OH Level Dichotomized	Count	Proportion
Normal	438	0.65276
Abnormal	233	0.34724
Total	671	1
**K: Dichotomized Vitamin D 1,25 Level Demographics for Subjects (61.4±1.1; n = 585; moderately right skewed distribution)**		
Vitamin D 1,25 Level Dichotomized	Count	Proportion
Normal	342	0.58462
Abnormal	243	0.41538
Total	585	1
**L: Dichotomized % Free Testosterone Level Demographics for Subjects (0.8±0.1; n = 711; strongly right skewed distribution)**		
% Free Testosterone Level Dichotomized	Count	Proportion
Normal	694	0.97609
Abnormal	17	0.02391
Total	711	1
**M: Dichotomized Testosterone Total Level Demographics for Subjects (30.8±1.5; n = 715; strongly right skewed distribution)**		
Testosterone Total Level Dichotomized	Count	Proportion
Normal	547	0.76503
Abnormal	168	0.23497
Total	715	1
**N: Dichotomized Estradiol Level Demographics for Subjects (97.4±7.4; n = 720; strongly right skewed distribution)**		
Estradiol Level Dichotomized	Count	Proportion
Pre-menopausal	631	0.87639
Post-menopausal	89	0.12361
Total	720	1
**O: Dichotomized Estrone Level Demographics for Subjects (69.7±4.2; n = 552; strongly right skewed distribution)**		
Estrone Level Dichotomized	Count	Proportion
Pre-menopausal	354	0.6413
Post-menopausal	198	0.3587
Total	552	1
**P: Dichotomized Estriol Level Demographics for Subjects (0.2±0.0; n = 513)**		
Estriol Level Dichotomized	Count	Proportion
Normal	1	0.00195
Abnormal	512	0.99805
Total	513	1
**Q: Dichotomized Beam Level Demographics for Subjects**		
Beam Level	Count	Proportion
Yes	192	0.2409
No	605	0.7591
Total	797	1
**R: Dichotomized P300 Speed (Latency) Level Demographics for Subjects (319.7±1.9; n = 194) moderately normal distribution)**		
P300 Speed (Latency) Level Dichotomized	Count	Proportion
Normal	90	0.46392
Abnormal	104	0.53608
Total	194	1
**S: Dichotomized P300 Voltage (Amplitude) Level Demographics for Subjects (4.8±0.4; n = 194; strongly right skewed distribution)**		
P300 Voltage (Amplitude) Level Dichotomized	Count	Proportion
Normal	5	0.02577
Abnormal	189	0.97423
Total	194	1

### Statistical Analysis

In order to examine the relationships among the variables, each and every pair of response and explanatory variables were examined using contingency analysis, bivariate scatter plots, simple linear regressions, as well as one-way analysis of variance (ANOVA) or Welch's t-test for classification variables. When the test of multiple means turned out to be statistically significant at 5% level, all pairs of means were compared using Tukey-Kramer's honestly significant difference as a post-hoc method.

Pearson product-moment correlations were calculated between hormone levels and the 12 mean domain scores, the total number of endorsed systems, and the grand mean across all 64 questions of the Menopause Questionnaire. A one-way ANOVA was performed for the origin of menopause variable for each of the 12 symptom domains, with a Bonferroni correction of p<.004 required for significance. Given the large number of domains and likely high inter-correlations between them, a factor analysis with principal components extraction and varimax rotation was performed on the 12 mean domain scores. Factor scores were generated for each patient and entered into the correlation analysis with the hormone levels. A similar factor analysis was performed on the hormone levels to reduce redundancy of highly inter-correlated values. Regression analyses were performed to predict the symptom domain score factors from the 15 hormone levels and again using the hormone level factors.

### EEG, P300, and Evoked Potentials Data

Lexicor and Cognitrace were used to determine P300 potential. A total of twenty electrodes were utilized: 3 along the central sulcus, 3 in the parietal region, 2 in the parietal/temporal region, 3 in the occipital region, 2 in the frontal/temporal region, as well as 5 in the frontal region. Calibration of the two machines was completed by repeat scans. Auditory stimuli of high and low beeps are utilized by Lexicor and Cognitrace with an output of latency and amplitude, dependent on age-based preprogrammed baselines. Listed in the patients' charts and determined through computer algorithm, latency (in milliseconds) and amplitude (in microvolts) from the waveform were selected for analysis.

## Results

### Preliminary Analyses

The detailed results of these preliminary analyses are not shown here due to the length, but they were used in constructing more elaborate multiple regression models and interpreting the results in the Corrected Analysis Section. In particular, the following tables summarize the statistical association of certain variables of interest.

### Association between Dichotomized FSH and P300 Speed/Voltage

Table 2 outlines the association between FSH and P300 speed/voltage. Latency was correlated with FSH (p = .02), but the fit was very poor (R2 = .02). P300 speed (dichotomized latency) is shown to be uncorrelated with FSH (p = .10). Amplitude or P300 voltage was also uncorrelated with FSH (p = .19).

**Table 2:** Association between FSH and P300 Speed/Voltage.

Test	Adjusted R2	N	F	P-Value	Chi2
Latency fitted by FSH (Bivariate)	0.02662	186	6.0595	0.0148*	-
P300 Speed fitted by FSH (Logistic)	-	-	-	0.162	1.95563
Amplitude fitted by FSH (Bivariate)	−0.00069	186	0.873	0.3513	-
P300 Voltage fitted by FSH (Logisitic)	-	-	-	0.0759	3.150917
Latency fitted by Dichotomozied FSH (ANOVA)	0.01976	186	4.7293	0.0309* (two-sided) 0.0155* (one-sided)	-
P300 Speed fitted by Dichotomized FSH (Contigency) Likelihood Ratio	-	-	-	0.0972	2.75
P300 Speed fitted by Dichotomized FSH (Contigency) Pearson	-	-	-	0.0977	2.742
P300 Speed fitted by Dichotomized FSH (Contigency) Fisher's Exact Test	-	-	-	-	0.1075
Amplitude fitted by Dichotomized FSH (ANOVA)	0.000838	186	1.1551	0.2839 (two-sided)	-
P300 Voltage fitted by Dichotomized FSH (Contingency) Likelihood Ratio	-	-	-	0.1895	1.721
P300 Voltage fitted by Dichotomized FSH (Contingency) Pearson	-	-	-	0.2063	1.597
P300 Voltage fitted by Dichotomized FSH (Contingency) Fisher's Exact Test	-	-	-	0.3705	-

### Association between Categorized FSH and P300 Speed/Voltage

Table 3 outlines the association between categorized FSH range and P300 latency/amplitude (i.e., speed/voltage). P300 speed was uncorrelated with FSH over 40 mIU/mL (or under 40 mIU/mL) (p = .27). Amplitude or P300 voltage also was uncorrelated with FSH over 40 mIU/mL (or under 40 mIU/mL) (p = .18). P300 speed was uncorrelated with FSH over 80 mIU/mL (or under 80 mIU/mL) (p = .65). P300 voltage also was uncorrelated with FSH over 80 mIU/mL (or under 80 mIU/mL) (p = .20). P300 speed was uncorrelated with FSH over 100 mIU/mL (or under 100 mIU/mL) (p = .13). P300 voltage also was uncorrelated with FSH over 100 mIU/mL (or under 100 mIU/mL) (p = .32).

**Table 3:** Association between Categorized FSH and P300 Speed/Voltage.

Test	Adjusted R2	N	F	t	P-Value	Chi2
Latency fitted by FSH over 40 (ANOVA)	0.019772	186	4.7316	2.17523	0.0309* (two sided) 0.0154* (one-sided)	-
P300 Speed fitted by FSH over 40 (Contingency) Likelihood Ratio	-	-	-	-	0.2678	1.228
P300 Speed fitted by FSH over 40 (Contingency) Pearson	-	-	-	-	0.2689	1.223
P300 Speed fitted by FSH over 40 (Contingency) Fisher's Exact Test	-	-	-	-	0.2785	-
Amplitude fitted by FSH over 40 (ANOVA)	0.00413	186	1.7671	-	0.1854 (two-sided)	-
P300 Voltage fitted by FSH over 40 (Contingency) Likelihood Ratio	-	-	-	-	0.0444*	4.041
P300 Voltage fitted by FSH over 40 (Contingency) Pearson	-	-	-	-	0.1133	2.507
P300 Voltage fitted by FSH over 40 (Contingency) Fisher's Exact Test	-	-	-	-	0.174	-
Latency fitted by FSH over 80 (ANOVA)	0.007259	186	2.3527	-	0.1268 (two-sided)	-
P300 Speed fitted by FSH over 80 (Contingency) Likelihood Ratio	-	-	-	-	0.6516	0.204
P300 Speed fitted by FSH over 80 (Contingency) Pearson	-	-	-	-	0.6522	0.203
P300 Speed fitted by FSH over 80 (Contingency) Fisher's Exact Test	-	-	-	-	0.6862	-
Amplitude fitted by FSH over 80 (ANOVA)	-0.00327	186	0.3977	-	0.5291 (two-sided)	-
P300 Voltage fitted by FSH over 80 (Contingency) Likelihood Ratio	-	-	-	-	0.1982	1.656
P300 Voltage fitted by FSH over 80 (Contingency) Pearson	-	-	-	-	0.34	0.911
Latency fitted by FSH over 100 (ANOVA)	0.031206	186	6.9591	2.638016	0.0091* (two-sided) 0.0045* (one-sided)	-
P300 Speed fitted by FSH over 100 (Contingency) Likelihood Ratio	-	-	-	-	0.1258	2.343
P300 Speed fitted by FSH over 100 (Contingency) Pearson	-	-	-	-	0.1323	2.266
P300 Speed fitted by FSH over 100 (Contingency) Fisher's Exact test	-	-	-	-	0.2015	-
Amplitude fitted by FSH over 100 (ANOVA)	−0.0054	186	0.0061	-	0.9376 (two-sided)	-
P300 Voltage fitted by FSH over 100 (Contingency) Likelihood Ratio	-	-	-	-	0.3241	0.972
P300 Voltage fitted by FSH over 100 (Contingency) Pearson	-	-	-	-	0.4722	0.517

### Association between FSH and P300 Speed/Voltage for FSH under/over 80

Tables 4 and 5 outline the associations between FSH range and P300 speed/voltage for FSH under/over 80 mIU/mL. Again, P300 speed was uncorrelated with FSH for the group with FSH under 80 mIU/mL (p = .12). P300 Voltage also was uncorrelated with FSH for the group with FSH under 80 mIU/mL (p = .21). Similarly, P300 speed was uncorrelated with FSH for the group with FSH over 80 mIU/mL (p = .36). Amplitude also was uncorrelated with FSH for the group with FSH over 80 mIU/mL (p = .10).

**Table 4:** Association between FSH and P300 Speed/Voltage for FSH under 80.

Test	Adjusted R2	N	F	P-value	Chi2
Latency fitted by FSH (bivariate)	0.015967	158	3.5476	0.0615	-
P300 Speed fitted by FSH (logistic)	-	-	-	0.1212	2.402083
Amplitude fitted by FSH (bivariate)	0.000377	158	1.0592	0.305	-
P300 Voltage fitted by FSH (logistic)	-	-	-	0.2116	1.560134

**Table 5:** Association between FSH and P300 Speed/Voltage for FSH over 80.

Test	Adjusted R2	N	F	P-value	Chi2
Latency fitted by FSH (bivariate)	−0.00906	28	0.7575	0.3921	-
P300 Speed fitted by FSH (logistic)	-	-	-	0.3595	0.839701
Amplitude fitted by FSH (bivariate)	0.063924	28	2.8438	0.1037	-

### Association between FSH and P300 Speed/Voltage for FSH under/over 100

Tables 6 and 7 outline the associations between FSH range and P300 speed/voltage for FSH under/over 100 mIU/mL. Again, P300 speed was uncorrelated with FSH for the group with FSH under 100 mIU/mL (p = .29). P300 voltage also was uncorrelated with FSH for the group with FSH under 100 mIU/mL (p = .13). Similarly, P300 speed was uncorrelated with FSH for the group with FSH over 100 mIU/mL (p = .63). Amplitude also was uncorrelated with FSH for the group with FSH over 100 mIU/mL (p = .51).

**Table 6:** Association between FSH and P300 Speed/Voltage for FSH under 100.

Test	Adjusted R2	N	F	P-value	Chi2
Latency fitted by FSH (bivariate)	0.000685	169	1.1151	0.2925	-
P300 Speed fitted by FSH (logistic)	-	-	-	0.5632	0.33424
Amplitude fitted by FSH (bivariate)	0.005156	169	1.8706	0.1732	-
P300 Voltage fitted by FSH (logistic)	-	-	-	0.1379	2.200685

**Table 7:** Association between FSH and P300 Speed/Voltage for FSH over 100.

Test	Adjusted R2	N	F	P-value	Chi2
Latency fitted by FSH (bivariate)	−0.04976	17	0.2416	0.6302	-
P300 Speed fitted by FSH (logistic)	-	-	-	0.6857	0.163758
Amplitude fitted by FSH (bivariate)	−0.03501	17	0.4587	0.5085	-

### Association between FSH over 40 and Estradiol, Estrone, and Estriol

Table 8 outlines the associations between FSH over 40 mIU/mL and estradiol, estrone and estriol. There was a strong association between estradiol and FSH over 40 mIU/mL (or under 40 mIU/mL) (p<.0001). More explicitly, estradiol level was significantly higher for the group with FSH under 40 mIU/mL (126.0±8.6 pg/mL) than for the group with FSH over 40 mIU/mL (27.1±13.5 pg/mL) (p<.0001). There was a higher percentage of patients with the pre-menopausal level of estradiol in the group with FSH under 40 mIU/mL than in the group with FSH over 40 mIU/mL (p<.0001).

**Table 8:** Association between FSH over 40 and Estradiol, Estrone, Estriol.

Test	Adjusted R2	N	F	t	P-value	Average	Std. Error	Lower 95%	Upper 95%	Chi2
Estradiol fitted by FSH over 40 (ANOVA)	0.04977	713	38.2927	−6.18811	<.0001* (two-sided) <.0001* (one-sided)	-	-	-	-	-
Estradiol fitted by FSH over 40	-	206	-	-	-	27.092	13.48	0.63	53.56	-
Estradiol fitted by FSH under 40	-	507	-	-	-	126.01	8.592	109.14	142.88	-
Dichotomized Estradiol fitted by FSH over 40 (Contingency) Likelihood Ratio	-	-	-	-	<.0001*	-	-	-	-	100.562
Dichotomized Estradiol fitted by FSH over 40 (Contingency) Pearson	-	-	-	-	<.0001*	-	-	-	-	111.681
Dichotomized Estradiol fitted by FSH over 40 (Contingency) Fisher's Exact Test	-	-	-	-	<.0001*	-	-	-	-	-
Estrone fitted by FSH over 40 (ANOVA)	0.036116	546	21.4206	−4.62824	<.0001* (two-sided) <.0001* (one-sided)					
Estrone fitted by FSH over 40	-	168	-	-	-	41.0796	7.5164	26.315	55.844	-
Estrone fitted by FSH under 40	-	378	-	-	-	82.8889	5.0109	73.046	92.732	-
Dichotomized Estrone fitted by FSH over 40 (Contingency) Likelihood Ratio	-	-	-	-	<.0001*	-	-	-	-	51.146
Dichotomized Estrone fitted by FSH over 40 (Contingency) Pearson	-	-	-	-	<.0001*	-	-	-	-	52.243
Dichotomized Estrone fitted by FSH over 40 (Contingency) Fisher's Exact Test	-	-	-	-	<.0001*	-	-	-	-	-
Estriol fitted by FSH over 40 (ANOVA)	0.005501	507	3.7991	1.949129	0.0518 (two-sided) 0.0259* (one-sided)	-	-	-	-	-
Estriol fitted by FSH over 40	-	152	-	-	-	0.19625	0.00776	0.181	0.2115	-
Estriol fitted by FSH under 40	-	355	-	-	-	0.178169	0.00508	0.16819	0.18815	-
Dichotomized Estriol fitted by FSH over 40 (Contingency) Likelihood Ratio	-	-	-	-	0.1203	-	-	-	-	2.414
Dichotomized Estriol fitted by FSH over 40 (Contingency) Pearson	-	-	-	-	0.1261	-	-	-	-	2.34
Dichotomized Estriol fitted by FSH over 40 (Contingency) Fisher's Exact Test	-	-	-	-	0.2998	-	-	-	-	-

Similarly, there was a strong association between estrone and FSH over 40 mIU/mL (or under 40 mIU/mL) (p<.0001). More explicitly, estrone level was significantly higher for the group with FSH under 40 mIU/mL (82.9±5.0 pg/mL) than for the group with FSH over 40 mIU/mL (41.1±7.5 pg/mL) (p<.0001). There was a higher percentage of patients with the pre-menopausal level of estrone in the group with FSH under 40 mIU/mL than in the group with FSH over 40 mIU/mL (p<.0001).

There was a weak association between estriol and FSH over 40 mIU/mL (or under 40 mIU/mL) (p = .03). Estriol level appeared to be slightly higher for the group with FSH over 40 mIU/mL (0.20±0.01 ng/mL) than for the group with FSH under 40 mIU/mL (0.18±0.01 ng/mL). After dichotomization, however, menopausal level of estriol was found to be uncorrelated with FSH over 40 mIU/mL (or under 40 mIU/mL) (p = .12).

Tables 9 and 10 outline the association between FSH and estradiol, estrone, and estriol for FSH under and over 40 mIU/mL. Estriol was uncorrelated with FSH for the group with FSH under 40 mIU/mL (p = .61). Estriol was uncorrelated with FSH for the group with FSH over 40 mIU/mL either (p = .92). Estradiol was strongly associated with FSH for the group with FSH over 40 mIU/mL (p<.0001). Estrone also was associated with FSH for the group with FSH over 40 mIU/mL (p<.01).

**Table 9:** Association between FSH and Estradiol, Estrone, Estriol for FSH under 40.

Test	Adjusted R2	N	F	P-value	Chi2
Estradiol fitted by FSH (bivariate)	0.013439	507	7.8927	0.0052*	-
Dichotomized Estradiol fitted by FSH (logistic)	-	-	-	0.57	0.322627
Estrone fitted by FSH (bivariate)	0.008586	378	4.2649	0.0396*	-
Dichotomized Estrone fitted by FSH (logistic)	-	-	-	0.0283*	4.812526
Estriol fitted by FSH (bivariate)	−0.00209	355	0.2614	0.6094	-

**Table 10:** Association between FSH and Estradiol, Estrone, Estriol for FSH over 40.

Test	Adjusted R2	N	F	P-value	Chi2
Estradiol fitted by FSH (bivariate)	0.13044	206	31.7514	<.0001*	-
Dichotomized Estradiol fitted by FSH (logistic)	-	-	-	<.0001*	30.51419
Estrone fitted by FSH (bivariate)	0.019264	168	4.2803	0.0401*	-
Dichotomized Estrone fitted by FSH (logistic)	-	-	-	0.0035*	8.534631
Estriol fitted by FSH (bivariate)	−0.00661	152	0.009	0.9245	-
Dichotomized Estriol fitted by FSH (logistic)	-	-	-	0.9758	0.000924

### Association between Leptin and P300 Speed/Voltage

Table 11 outlines the association between leptin and P300 speed/voltage. High leptin levels were determined as>5 ng/mL and high P300 latencies were bdetermined by a patient's age+300 ms (i.e., a patient age 50 should have a P300 latency of 350, so any value greater than 350, shows a delay in latency). P300 speed appeared to be correlated with leptin (p = .01), with higher leptin level associated with abnormal P300 speed (OR = 0.97). However, once dichotomized, this association was insignificant (p = .10). Amplitude or P300 voltage was uncorrelated with leptin (p>.30). Furthermore, higher leptin levels are indicative of obesity and its risks and delayed latency shows slowed brain speed and attention.

**Table 11:** Association between Leptin and P300 Speed/Voltage.

Test	Adjusted R2	N	F	P-value	Chi2
Latency fitted by Leptin (bivariate)	0.048107	159	8.985	0.0032*	-
P300 Speed fitted by Leptin (logistic)	-	-	-	0.0083*	6.972178
Amplitude fitted by Leptin (bivariate)	−0.00036	159	0.9426	0.3331	-
P300 Voltage fitted by Leptin (logistic)	-	-	-	0.7599	0.09338
Latency fitted by Dichotomized Leptin (ANOVA)	0.000685	159	1.1083	0.2941 (two-sided)	-
P300 Speed fitted by Dichotomized Leptin (Contingency) Likelihood Ratio	-	-	-	0.1014	2.683
P300 Speed fitted by Dichotomized Leptin (Contingency) Pearson	-	-	-	0.1018	2.677
P300 Speed fitted by Dichotomized Leptin (Contingency) Fisher's Exact Test	-	-	-	0.1131	-
Amplitude fitted by Dichotomized Leptin (ANOVA)	−0.00364	159	0.4273	0.5143 (two-sided)	-
P300 Voltage fitted by Dichotomized Leptin (Contingency) Likelihood Ratio	-	-	-	0.8087	0.059
P300 Voltage fitted by Dichotomized Leptin (Contingency) Pearson	-	-	-	0.8094	0.058

### Regression Analysis

In order to assess the pattern of each hormone level on P300 speed/voltage accounting for any (additive) effect of covariate measured in this study such as age, multiple regression models were constructed with P300 speed/voltage as response variables. Final models were then built by reducing the full models through a stepwise regression method. Only the main effects were considered in the model, and dependency among the response variables was not considered for an individual examination of each response variable. The following tables and figures summarize the results of the reduced model fit, ANOVA, and effect estimates, along with prediction profiler of main effects in the reduced model.

### Latency predicted by Leptin with Age (ANOVA) and P300 Speed predicted by Leptin with Age (Logistic)

**Figure 2:**
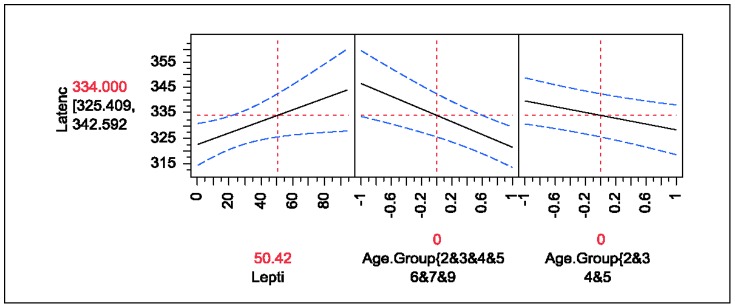
Prediction Profiler for Latency predicted by Leptin with Age (ANOVA).

Once age adjusted, leptin was found to be the only statistically significant predictor for latency or P300 amplitude (p = .03), with the effect size of 0.23. Hence, the higher the leptin level, the longer the latency. After age adjustment, higher leptin level was more associated with abnormal P300 latency (OR = 0.98). Age alone also was a statistically significant factor for latency or P300 speed (p<.01): the older the patient, the longer was the latency. However, even after age adjustment, amplitude and P300 voltage were found to be uncorrelated with leptin (p>.25). Also, once age adjusted, none of FSH, LH, or TSH were found to be statistically significant predictors of the P300 measures (p>.25) (see Tables 12–16 and Figure 2).

**Table 12:** Latency Variables predicted by Leptin with Age (ANOVA).

Test	R2	Adjusted R2	N	F	P-value
Latency predicted by Leptin with Age (ANOVA)	0.1573	0.140884	158	9.582	<.0001*

**Table 13:** Regression Analysis of Latency predicted by Leptin with Age (ANOVA).

Parameter Estimates Term	Estimate	Std.Error	t	P-value	Lower 95%	Upper 95%
Intercept	322.37092	4.244617	75.95	<.0001*	313.98573	330.75611
Leptin	0.2306531	0.10853	2.13	0.0352*	0.0162537	0.4450526
Age Group: 2&3&4&5–6&7&9	−12.66445	3.382663	−3.74	0.0003*	−19.34686	−5.982038
Age Group: 2&3–4&5	−5.758462	2.11586	−2.72	0.0072*	−9.938319	−1.578605

**Table 14:** P300 Speed Variables predicted by Leptin with Age (logistic).

Test	N	Chi2	P-value
P300 Speed predicted by Leptin with Age(logistic)	158	18.57717	<.0001*

**Table 15:** Regression Analysis of P300 Speed predicted by Leptin with Age (logistic).

Parameter Estimates Term	Estimate	Std.Error	t	P-value	Lower 95%	Upper 95%
Intercept	−0.5940827	0.2620591	5.14	0.0234*	−1.1230387	−0.0913201
Leptin	0.02216648	0.0100457	4.87	0.0273*	0.00320555	0.04286365
Age Group: 2&3–4&5&6&7&9	−0.6206882	0.1834263	11.45	0.0007*	−0.9905093	−0.2683189

**Table 16:** P300 Speed Odds Ratio predicted by Leptin with Age (logistic).

Odd Ratios Term	Odds Ratio	Lower 95%	Upper 95%	Reciprocal
Leptin	1.022414	1.003211	1.043796	0.9780774
Age Group: 2&3–4&5&6&7&9	0.537574	0.371387	0.764664	1.8602078

### Principal Component Analysis

The principal component analysis (factor analysis) was performed to derive a small number of independent linear combinations (viz., principal components) of a set of variables that capture as much of the variability in the original variables as possible. Reducing the dimensionalities of this multivariate data, the most important components were used to visualize the structure of the data. Each principal component was calculated by taking a linear combination of an eigenvector of the correlation matrix with a variable. The corresponding eigenvalues were the variance of each component.

### Principal Component Analysis in the Raw Scale

**Figure 3:**
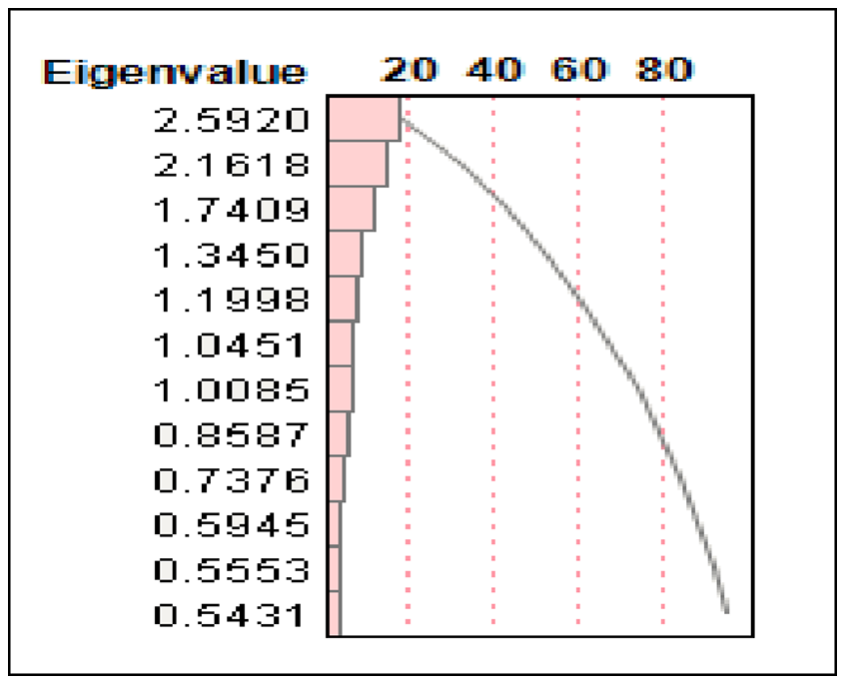
Principal Component Analysis in the Raw Scale Eigenvalue Plot.

**Figure 4:**
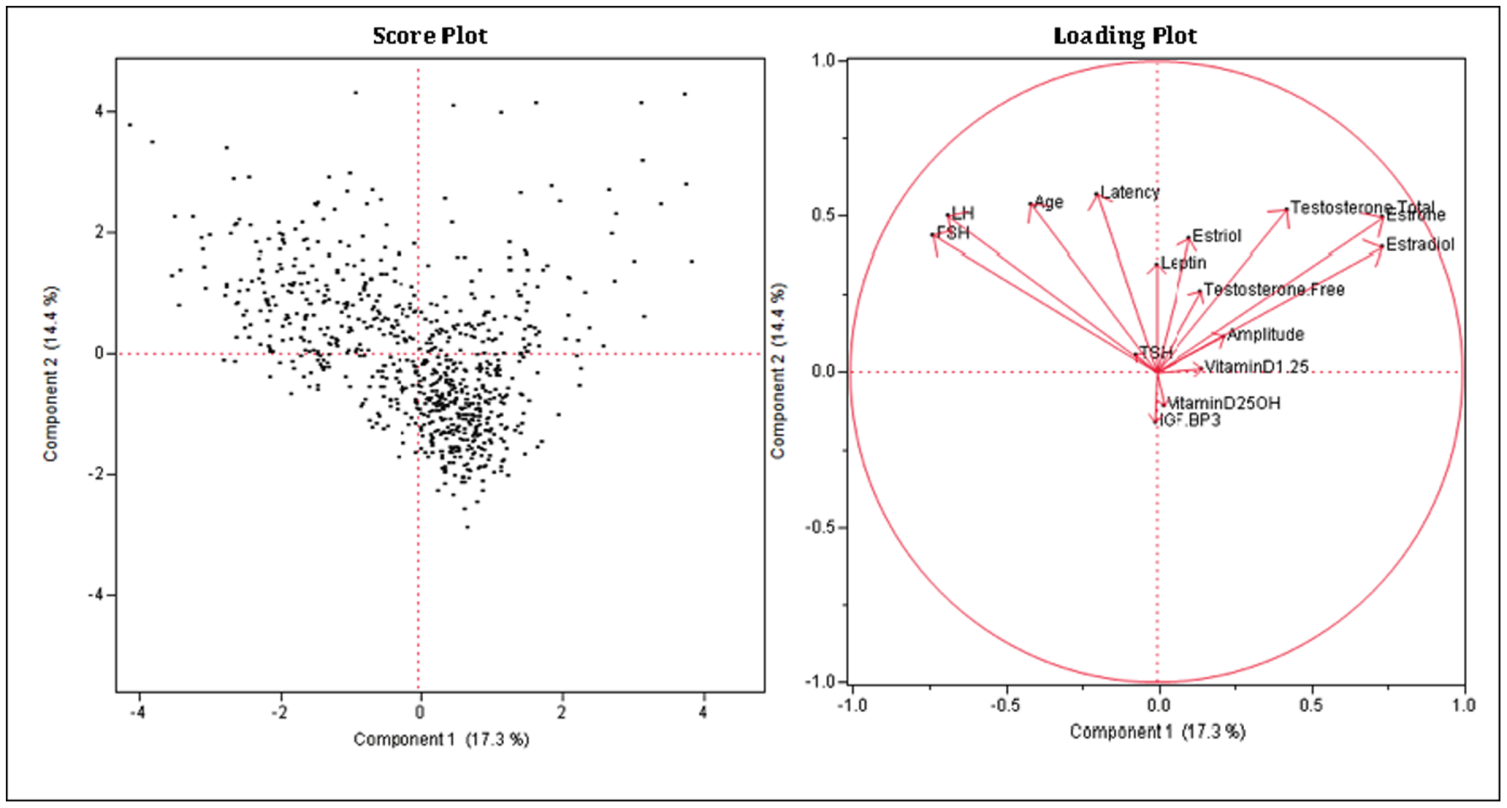
Principal Component Analysis in the Raw Scale Score and Loading Plot.

The proportion of variation explained by clustering was 0.413. The first two principal components accounted for 31.7% of the variability while the first three components accounted for 43.3% of the variability. Based on these components, clustering of the variables was done producing three clusters, each with five variables. There was no distinct or definite association found among the variables (at least the ones being examined). One of the causes was the incompleteness of the data, with many missing values, along with the retrospective aspect of the study. Although some variables tended to have a sizable R2, those strongly right skewed variables from the raw data in a continuous scale were log-transformed to re-perform the principal component analysis to determine possible additional results (see Tables 17–21; Figures 3, 4).

**Table 17:** Principal Component Analysis in the Raw Scale Correlations Estimated by Pairwise Method.

	Age	FSH	LH	TSH	Leptin	IGF-BP3	Vitamin D 25OH	Vitamin D 1,25	Testosterone Free	Testosterone Total	Estradiol	Estrone	Estriol	P300 Latency	P300 Amplitude
Age	1	0.4089	0.3833	0.0212	0.0491	−0.1876	0.0285	0.0185	0.0456	0.0471	−0.0623	−0.0243	0.0475	0.3349	−0.0062
FSH	0.4089	1	0.855	0.0188	−0.0065	0.0676	0.0051	0.0013	−0.0282	−0.0848	−0.2378	−0.2036	0.0685	0.1786	−0.0687
LH	0.3833	0.855	1	0.0456	0.0531	0.0337	0.0213	−0.0252	0.0135	−0.0431	−0.159	−0.1531	0.0635	0.2161	−0.0605
TSH	0.0212	0.0188	0.0456	1	0.0861	−0.1212	−0.0426	−0.1096	−0.0099	−0.0109	−0.0168	−0.0229	−0.0505	0.0286	0.0314
Leptin	0.0491	−0.0065	0.0531	0.0861	1	0.0089	−0.3006	−0.2316	0.0141	0.1042	−0.0135	0.0942	0.1898	0.2327	0.0773
IGF-BP3	−0.1876	0.0676	0.0337	−0.1212	0.0089	1	0.0375	0.1707	0.0553	0.0029	−0.1208	−0.0747	0.0538	−0.1793	0.1971
Vitamin D 25OH	0.0285	0.0051	0.0213	−0.0426	−0.3006	0.0375	1	0.4501	0.0149	0.0463	0.0211	−0.0437	−0.1422	−0.0048	0.0313
Vitamin D 1,25	0.0185	0.0013	−0.0252	−0.1096	−0.2316	0.1707	0.4501	1	0.0278	0.0514	0.1066	0.1357	0.0964	−0.0093	−0.0129
Testosterone Free	0.0456	−0.0282	0.0135	−0.0099	0.0141	0.0553	0.0149	0.0278	1	0.3713	0.0535	0.0678	0.0102	0.067	0.0027
Testosterone Total	0.0471	−0.0848	−0.0431	−0.0109	0.1042	0.0029	0.0463	0.0514	0.3713	1	0.3284	0.4243	0.1873	0.1572	−0.015
Estradiol	−0.0623	−0.2378	−0.159	−0.0168	−0.0135	−0.1208	0.0211	0.1066	0.0535	0.3284	1	0.8814	0.0845	−0.0129	0.1637
Estrone	−0.0243	−0.2036	−0.1531	−0.0229	0.0942	−0.0747	−0.0437	0.1357	0.0678	0.4243	0.8814	1	0.1776	0.0289	0.1819
Estriol	0.0475	0.0685	0.0635	−0.0505	0.1898	0.0538	−0.1422	0.0964	0.0102	0.1873	0.0845	0.1776	1	0.1752	0.1386
P300 Latency	0.3349	0.1786	0.2161	0.0286	0.2327	−0.1793	−0.0048	−0.0093	0.067	0.1572	−0.0129	0.0289	0.1752	1	−0.0019
P300 Amplitude	−0.0062	−0.0687	−0.0605	0.0314	0.0773	0.1971	0.0313	−0.0129	0.0027	−0.015	0.1637	0.1819	0.1386	−0.0019	1

**Table 18:** Principal Component Analysis in the Raw Scale Eigenvalues.

Number	Eigenvalue	Percent	Cumulative Percent	Chi2	DF	P-value
1	2.592	17.28	17.28	3675.13	104.201	<.0001*
2	2.1618	14.412	31.692	3088.95	94.006	<.0001*
3	1.7409	11.606	43.298	2585.9	83.647	<.0001*
4	1.345	8.967	52.265	2203.61	73.1	<.0001*
5	1.1998	7.999	60.264	1968.51	62.613	<.0001*
6	1.0451	6.967	67.231	1754.61	52.88	<.0001*
7	1.0085	6.724	73.955	1575.81	43.82	<.0001*
8	0.8587	5.725	79.679	1353.07	35.394	<.0001*
9	0.7376	4.918	84.597	1164.67	27.732	<.0001*
10	0.5945	3.963	88.56	998.46	20.907	<.0001*
11	0.5553	3.702	92.262	886.619	14.907	<.0001*
12	0.5431	3.621	95.883	736.788	9.894	<.0001*
13	0.3825	2.55	98.433	420.531	5.908	<.0001*
14	0.1394	0.93	99.363	27.714	2.852	<.0001*
15	0.0956	0.637	100	-	.	-

**Table 19:** Principal Component Analysis in the Raw Scale Variable Clustering Summary.

Cluster	Number of Members	Most Representative Variable	Proportion of Variation Explained
1	5	LH	0.453
2	5	Estrone	0.443
3	5	Vitamin D 25OH	0.342

**Table 20:** Principal Component Analysis in the Raw Scale Cluster Members.

Cluster	Members	R2 with Own Cluster	R2 with Next Closest	1-R2 Ratio
1	LH	0.779	0.021	0.226
	FSH	0.776	0.047	0.235
	Age	0.478	0	0.522
	Latency	0.223	0.015	0.789
	IGF-BP3	0.01	0.009	0.999
2	Estrone	0.856	0.019	0.147
	Estradiol	0.795	0.029	0.212
	Testosterone Total	0.42	0	0.58
	Testosterone Free	0.084	0	0.917
	Amplitude	0.063	0.004	0.941
3	Vitamin D 25OH	0.64	0	0.36
	Vitamin D 1,25	0.53	0.014	0.477
	Leptin	0.44	0.007	0.564
	TSH	0.051	0.002	0.951
	Estriol	0.05	0.032	0.982

**Table 21:** Principal Component Analysis in the Raw Scale Standardized Components.

Variable	Cluster 1	Cluster 2	Cluster 3
	Coefficients	Coefficients	Coefficients
Age	0.4593895	0	0
FSH	0.5852787	0	0
LH	0.5863316	0	0
TSH	0	0	−0.172013
Leptin	0	0	−0.506993
IGF-BP3	−0.065645	0	0
Vitamin D 25OH	0	0	0.6119382
Vitamin D 1,25	0	0	0.5566771
Testosterone Free	0	0.194114	0
Testosterone Total	0	0.4353115	0
Estradiol	0	0.598654	0
Estrone	0	0.6213278	0
Estriol	0	0	−0.170329
Latency	0.3135539	0	0
Amplitude	0	0.1684897	0

### Principal Component Analysis in the Log Scale

**Figure 5:**
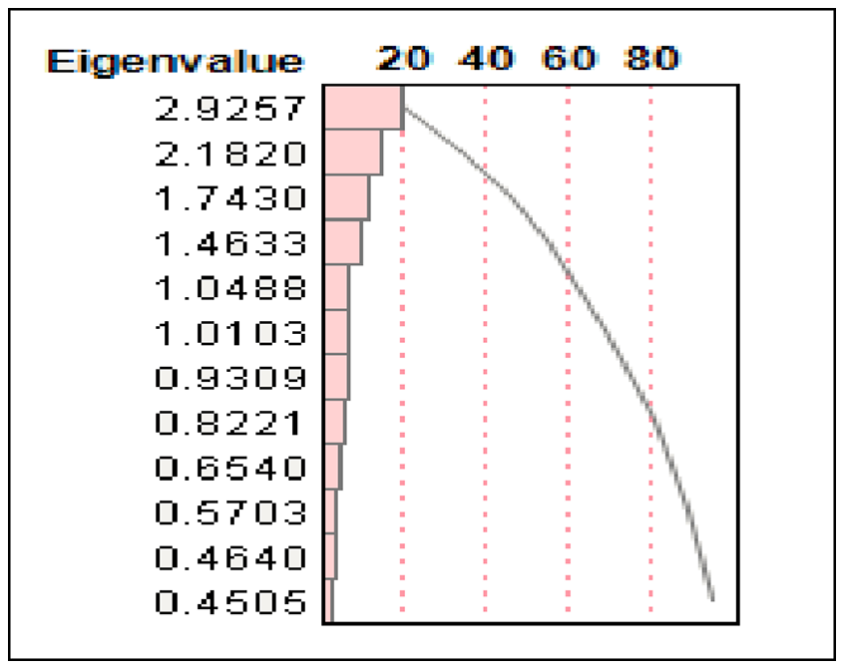
Principal Component Analysis in the Log Scale Eigenvalue Plot.

**Figure 6:**
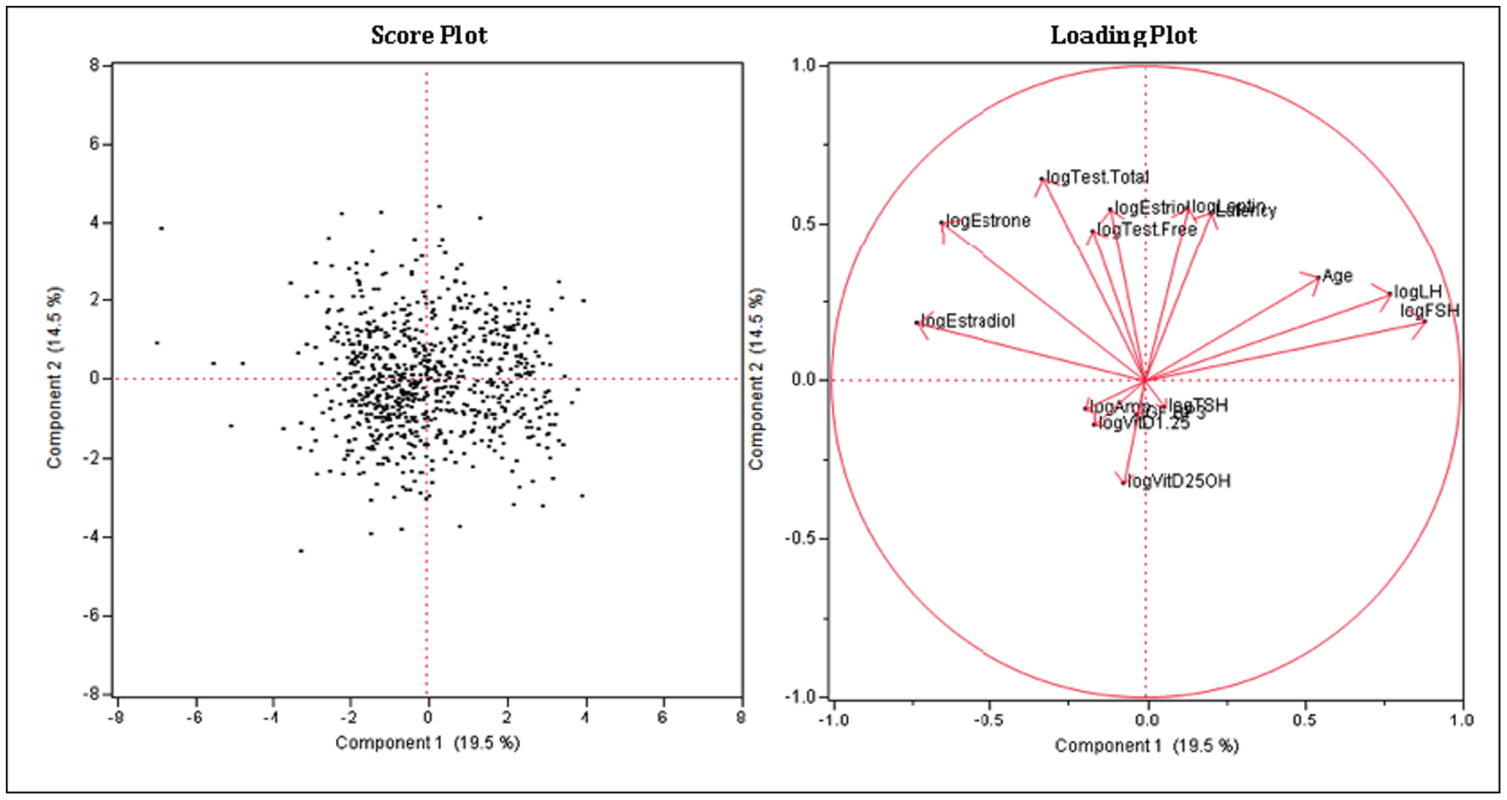
Principal Component Analysis in the Log Scale Score and Loading Plot.

The first two principal components accounted for 34.1% of the variability, while the first three components accounted for 45.7% of the variability, slightly better than the previous result in the raw scale. Based on these components, clustering of the variables was done producing five clusters, each with a varying number of variables. With an increased number of clusters, each cluster had even fewer associated variables. This confirmed the findings of no clear associations among the variables (among those being examined) and the results of regression analyses. Even in the log scale, some variables were still clustered together. FSH and LH were found to be associated while Vitamin D 1,25 and Vitamin D 25 OH tended to be together. Also, P300 latency and log leptin were found to be associated in the same cluster (see Tables 22–25; Figures 5, 6).

**Table 22:** Principal Component Analysis in the Log Scale Correlations Estimated by Pairwise Method.

	Age	log FSH	log LH	log TSH	log Leptin	IGF-BP3	log Vitamin D 25OH	log Vitamin D 1,25	log Testosterone Free	log Testosterone Total	log Estradiol	log Estrone	log Estriol	P300 Latency	log P300 Amplitude
Age	1	0.4881	0.409	−0.0145	0.0703	−0.1876	0.0427	0.0276	0.0577	0.0153	−0.2272	−0.1179	0.0348	0.3349	−0.0551
log FSH	0.4881	1	0.9034	−0.0002	0.0654	0.0219	−0.0343	−0.0615	−0.0069	−0.0804	−0.5312	−0.3933	0.0385	0.1488	−0.1109
log LH	0.409	0.9034	1	0.0128	0.1233	0.0134	−0.0507	−0.1047	0.0008	−0.0281	−0.2988	−0.2568	0.0559	0.1629	−0.086
log TSH	−0.0145	−0.0002	0.0128	1	0.0563	−0.1445	−0.0747	−0.0622	−0.0087	−0.1286	−0.0267	−0.0712	−0.038	−0.0535	0.0254
log Leptin	0.0703	0.0654	0.1233	0.0563	1	0.0368	−0.372	−0.2557	0.0666	0.1137	−0.0967	0.1198	0.2249	0.2878	−0.124
IGF-BP3	−0.1876	0.0219	0.0134	−0.1445	0.0368	1	0.0179	0.188	0.1104	0.0185	−0.1823	−0.0768	0.0919	−0.1793	0.256
log Vitamin D 25OH	0.0427	−0.0343	−0.0507	−0.0747	−0.372	0.0179	1	0.445	−0.0519	−0.0004	0.0534	−0.0968	−0.1206	0.0314	0.0362
log Vitamin D 1,25	0.0276	−0.0615	−0.1047	−0.0622	−0.2557	0.188	0.445	1	0.037	0.0988	0.0252	0.0454	0.1083	0.0168	0.0939
log Testosterone Free	0.0577	−0.0069	0.0008	−0.0087	0.0666	0.1104	−0.0519	0.037	1	0.4632	0.1064	0.1742	0.1082	0.0966	0.0816
log Testosterone Total	0.0153	−0.0804	−0.0281	−0.1286	0.1137	0.0185	−0.0004	0.0988	0.4632	1	0.2736	0.3963	0.2832	0.1846	0.0303
log Estradiol	−0.2272	−0.5312	−0.2988	−0.0267	−0.0967	−0.1823	0.0534	0.0252	0.1064	0.2736	1	0.6419	0.0133	−0.0557	0.0696
log Estrone	−0.1179	−0.3933	−0.2568	−0.0712	0.1198	−0.0768	−0.0968	0.0454	0.1742	0.3963	0.6419	1	0.3282	0.0728	0.0222
log Estriol	0.0348	0.0385	0.0559	−0.038	0.2249	0.0919	−0.1206	0.1083	0.1082	0.2832	0.0133	0.3282	1	0.1453	0.1198
P300 Latency	0.3349	0.1488	0.1629	−0.0535	0.2878	−0.1793	0.0314	0.0168	0.0966	0.1846	−0.0557	0.0728	0.1453	1	−0.1278
log P300 Amplitude	−0.0551	−0.1109	−0.086	0.0254	−0.124	0.256	0.0362	0.0939	0.0816	0.0303	0.0696	0.0222	0.1198	−0.1278	1

**Table 23:** Principal Component Analysis in the Log Scale Eigenvalues.

Number	Eigenvalue	Percent	Cumulative Percent	Chi2	DF	P-value
1	2.9257	19.505	19.505	4057.56	104.433	<.0001*
2	2.182	14.547	34.051	3265.1	94.786	<.0001*
3	1.743	11.62	45.672	2709.8	84.338	<.0001*
4	1.4633	9.755	55.427	2284.04	73.744	<.0001*
5	1.0488	6.992	62.419	1925.5	63.375	<.0001*
6	1.0103	6.736	69.154	1760.28	53.245	<.0001*
7	0.9309	6.206	75.36	1565.27	43.927	<.0001*
8	0.8221	5.481	80.841	1358.58	35.545	<.0001*
9	0.654	4.36	85.201	1160.88	27.874	<.0001*
10	0.5703	3.802	89.003	1035.24	21.008	<.0001*
11	0.464	3.093	92.097	924.225	14.962	<.0001*
12	0.4505	3.003	95.1	854.797	9.884	<.0001*
13	0.42	2.8	97.9	734.788	5.813	<.0001*
14	0.2629	1.753	99.653	464.47	2.814	<.0001*
15	0.0521	0.347	100	0	0.03	0.3172

**Table 24:** Principal Component Analysis in the Log Scale Cluster Members.

Cluster	Members	R2 with Own Cluster	R2 with Next Closest	1-R2 Ratio
1	log FSH	0.924	0.028	0.079
	log LH	0.775	0.032	0.233
	Age	0.419	0.064	0.62
	log Estradiol	0.379	0.153	0.734
	log Testosterone Total	0.673	0.035	0.339
2	log Testosterone Free	0.381	0.015	0.629
	log Estrone	0.484	0.186	0.634
	log Estriol	0.34	0.053	0.697
	log TSH	0.04	0.006	0.967
	log Vitamin D 1,25	0.723	0.032	0.287
3	log Vitamin D 25OH	0.723	0.045	0.291
	Latency	0.644	0.046	0.373
	log Leptin	0.644	0.136	0.412
	IGF-BP3	0.628	0.015	0.378
	Estriol	0.05	0.032	0.982

**Table 25:** Principal Component Analysis in the Log Scale Standardized Components.

Variable	Cluster 1 Coefficients	Cluster 2 Coefficients	Cluster 3 Coefficients
Age	0.4098452	0	0
log FSH	0.6082957	0	0
log LH	0.5570797	0	0
log TSH	0	−0.14372	0
log Leptin	0	0	0
IGF-BP3	0	0	0
log Vitamin D 25OH	0	0	0.7071068
log Vitamin D 1,25	0	0	0.7071068
log Testosterone Free	0	0.4456279	0
log Testosterone Total	0	0.5923475	0
log Estradiol	−0.389443	0	0
log Estrone	0	0.502436	0
log Estriol	0	0.4212397	0
Latency	0	0	0

### K-Means Clustering Analysis

**Figure 7:**
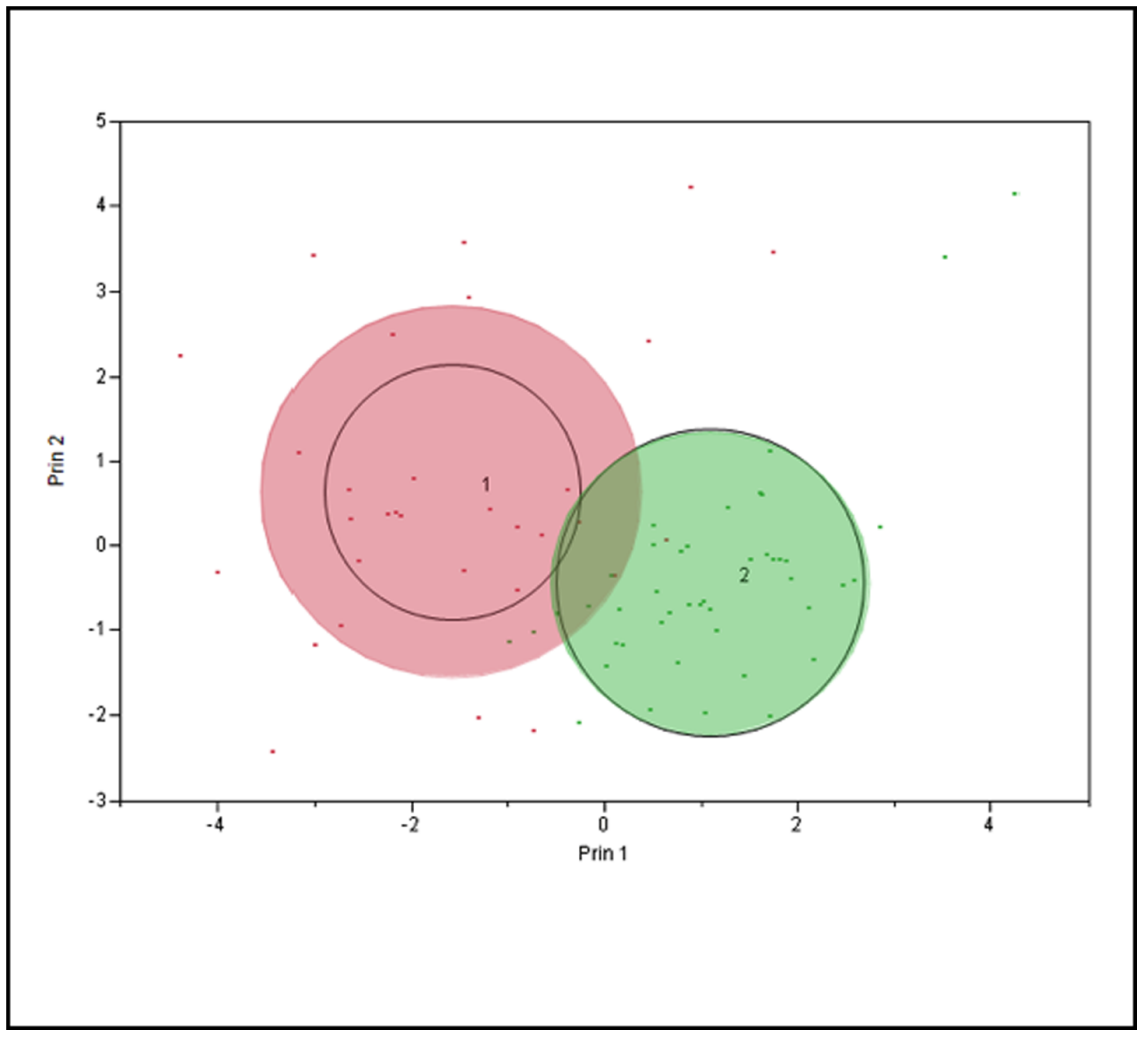
K-Means Clustering Analysis Cluster Bi-Plot.

**Figure 8:**
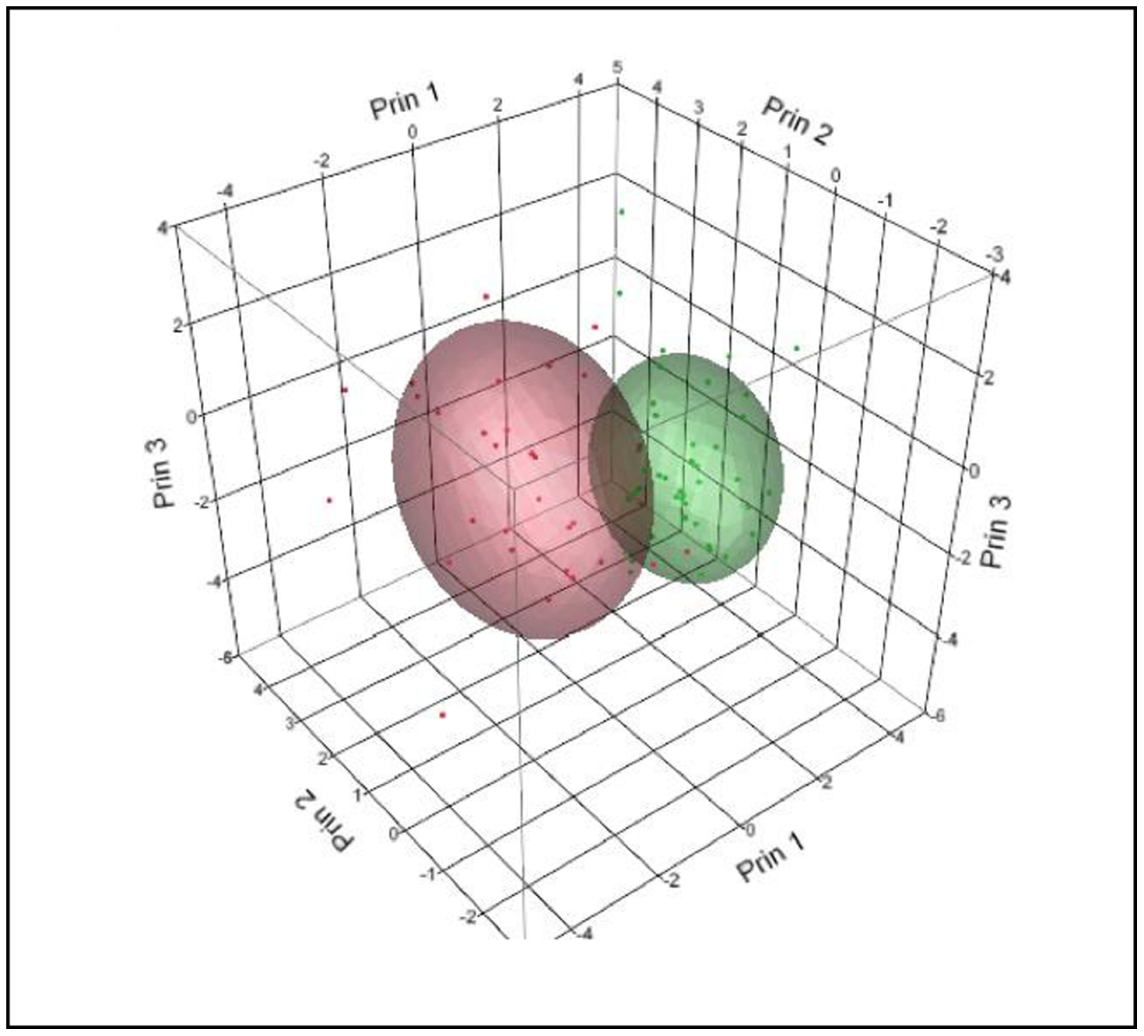
K-Means Clustering Analysis Cluster Bi-Plot in 3D.

**Figure 9:**
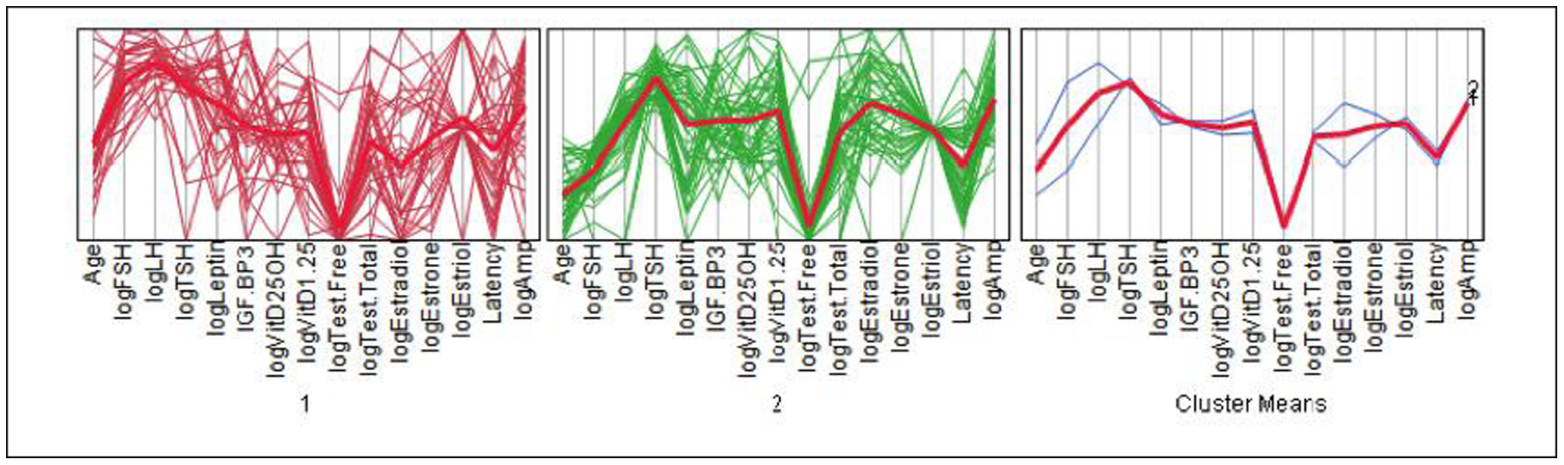
K-Means Clustering Analysis Parallel Coordinate Plot.

**Figure 10:**
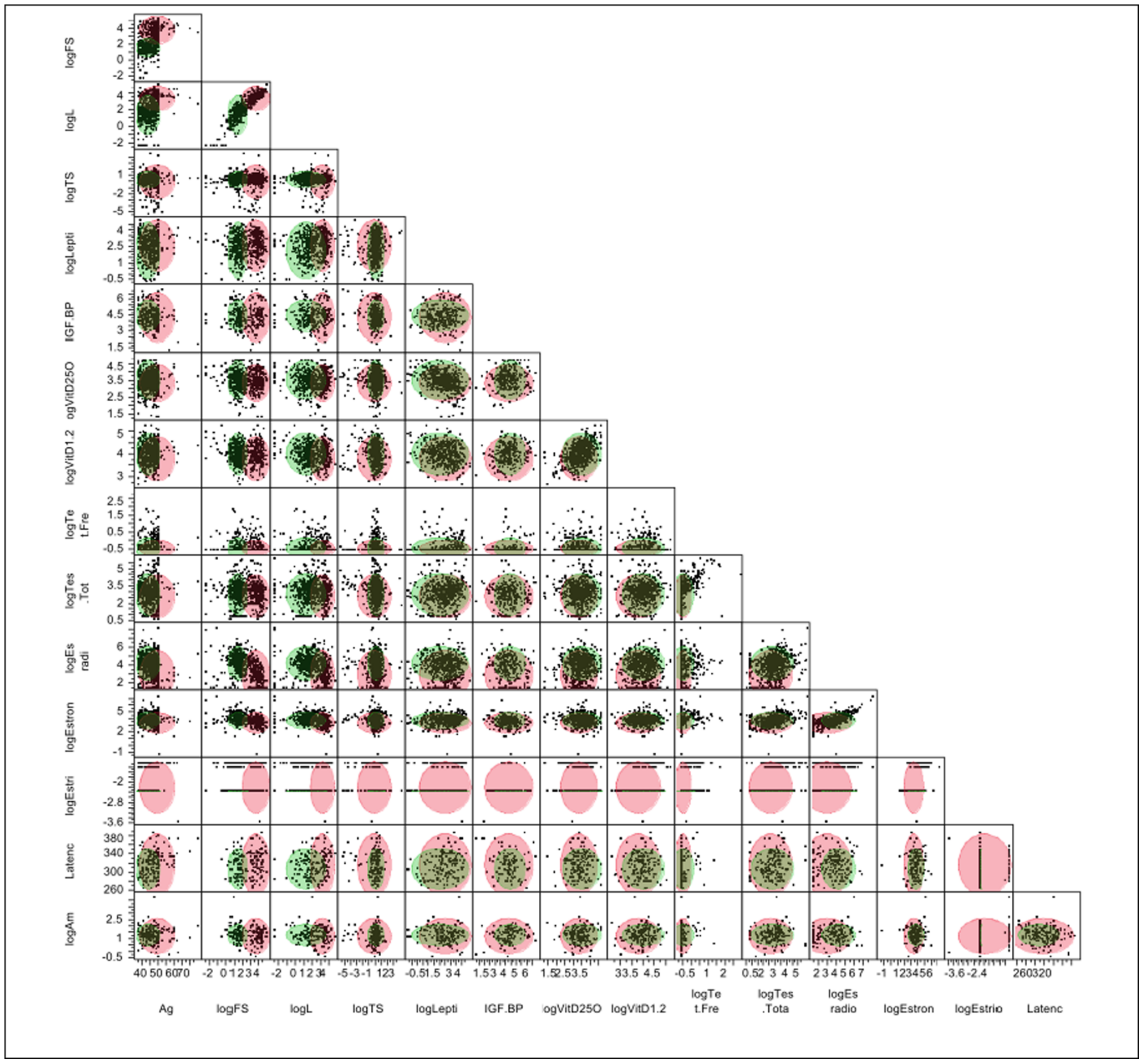
K-Means Clustering Analysis Scatter Plot Matrix.

As an alternative clustering analysis, the K-means clustering analysis with K = 2 was performed in the log scale of selected variables, which were strongly right skewed. Initially, the hierarchical clustering was performed to produce a tree-like classification structure, but no distinctive branches could be located to cluster the data in an effective way. In the K-means clustering, many different numbers of clusters were tried, but the two-means seemed to produce the most reasonable outcome, given the incompleteness of the dataset. The variables were scaled individually, and the within-cluster standard deviations were used producing cubic clustering criterion = −4.9793. Each cluster contained 31 and 45 points, respectively. The most representative variable separating these two clusters was log FSH, followed by log LH. The data resolution was found to be weak over other variables; see the scatterplot matrix below. With aforementioned reasons, nevertheless, the clustering was not highly successful with this dataset as the points were scattered without distinctive associations. Although the data were forced to cluster into two here, the parallel coordinate plots showed that the variable patterns were quite similar between the two groups, comparable to the overall cluster means, which resulted in low resolution of clustering (see Tables 26–30; Figures 7–10).

**Table 26:** K-Means Clustering Analysis Cluster Summary.

Cluster	Count
1	31
2	45

**Table 27:** K-Means Clustering Analysis Cluster Averages by Variable (Age+Specified Hormones).

Cluster	Age	log FSH	log LH	log TSH	log Leptin	IGF-BP3	log Vitamin D 25OH	log Vitamin D 1,25
1	50.4193548	3.84692834	3.33115457	0.07086968	2.62396293	4.40645161	3.37464635	3.83294885
2	44.4444444	1.60898738	1.40340109	0.37980236	2.14941452	4.52888889	3.55641941	4.05081038

**Table 28:** K-Means Clustering Analysis Cluster Averages by Variable (Specified Hormones+P300).

Cluster	log Testosterone Free	log Testosterone Total	log Estradiol	log Estrone	log Estriol	P300 Latency	log P300 Amplitude
1	−0.432203	2.6949935	3.08398269	3.52383408	−2.1642274	318.480645	1.28841661
2	−0.4176911	2.84516834	4.33986953	3.86399908	−2.3025851	309.948889	1.37825673

**Table 29:** K-Means Clustering Analysis Cluster Standard Deviations by Variable (Age+Specified Hormones).

Cluster	Age	log FSH	log LH	log TSH	log Leptin	IGF-BP3	log Vitamin D 25OH	log Vitamin D 1,25
1	5.13541903	0.81303646	0.67265226	1.2872815	1.09259949	1.06587401	0.50428182	0.45624656
2	3.49320328	0.53294581	1.08362395	0.63573686	1.20339793	0.68268171	0.53741888	0.43474834

**Table 30:** K-Means Clustering Analysis Cluster Standard Deviations by Variable (Specified Hormones+P300).

Cluster	log Testosterone Free	log Testosterone Total	log Estradiol	log Estrone	log Estriol	P300 Latency	log P300 Amplitude
1	0.21883302	0.87241656	1.24017776	0.6873002	0.47147219	31.8035443	0.64528174
2	0.28383649	0.85136274	0.87015612	0.56004314	1.33E-15	21.0254938	0.38876941

## Discussion

Although our data did not support a significant association between sex hormones and the electrophysiological parameters examined, most importantly, we did find a significant association between plasma increased leptin levels and P300 latency. Leptin was found to be the only statistically significant predictor for latency or P300 speed. Thus, the higher the leptin level was, the longer was the latency. After age adjustment, higher leptin level was associated more with abnormal P300 speed. While we found that age alone was a statistically significant factor for latency or P300 speed, we inferred that the older the patient was, the longer was the latency. However, even after age adjustment, amplitude and P300 voltage were found to be uncorrelated with leptin. Also, once age was adjusted, none of the FSH, LH, and TSH levels were found to be statistically significant predictors for P300 speed and voltage despite some research suggesting otherwise. In particular, Davis *et al.* [19] found that triiodothyronine (T_3_) levels were much lower in advanced AD Braak stages V–VI. Short *et al.* [15] also found significantly increased levels of LH and GRH in estrogen-free AD patients. In spite of our results, there is a historical positive correlation between the fluctuation in specific sex hormones (e.g. T3, LH, GRH, etc.) and advanced stages of AD, which should be further investigated in future studies elucidating such effects.

There is also significant evidence showing that P300 evoked potentials and neurophysiological tests validate PET measures of brain metabolism in cognitively impaired patients [73]. P300 has important clinical utility in that its measurements are representative of cognitive decline, especially those patients who are already suffering from progressive cognitive impairments or dementia. Reduced P300 amplitudes, delayed latencies, and neuropsychological deficits show positive correlations with PET brain hypometabolism; thus, the P300 serves as a cost-effective indicator that is essential in primary care settings and may help determine early abnormalities in cognition.

These findings may be useful in determining the role of leptin in future studies focusing on obesity and cognition. Certainly there is enough evidence to link leptin plasma levels as a function of cellular resistance and obesity [44], especially in menopausal women [45]. While this is fairly clear, less is known concerning the role of leptin and cognition.

There have been studies suggesting that obesity and excessive weight in middle aged adults is a risk factor for cognitive decline later in life [46]–[48]; congruently, observational studies of Alzheimer's Disease (AD) patients provides potential links between adipose tissue metabolism and cognition, weight loss occurring some years prior to the onset of clinical AD symptoms [49], [50]. The adipokine leptin, a cytokine secreted by adipose tissue, serves as an appetite suppressor and helps to regulate the body's energy expenditure–increased plasma levels of leptin associated with excessive body fat [51].

Mouse models have linked disrupted leptin receptors as potential sources for long-term potentiation impairments, synaptic plasticity issues, and deficiencies in spatial learning [52]. Furthermore, hippocampal viral vector introduction of leptin in rodents induced neuronal precursor proliferation and reduced neuro-degeneration; in addition, it suppressed memory consolidation and reduced the spatial awareness for food of administered rodents [53], [59]. The creation and removal of beta amyloid (Aβ) in rodents occurs through leptin modulation [54], [55], Aβ hippocampal removal was related to advanced object recognition [46]. In addition, following ischemic challenge, neuronal survival in the hippocampus for Gerbils treated with leptin was greater than for gerbils without leptin treatment [56]. Senescence-accelerated prone mice (SAMP8) with impaired learning and memory showed improved retention after hippocampal leptin injections [57]. Through such rodent modeling, much has been learnt about leptin and cognition.

In humans, there remains controversy regarding the role of leptin in cognition. Lieb *et al.* indicated that the presence of increased hippocampal and whole brain volume were associated with higher plasma levels of leptin, suggesting that these higher plasma leptin levels associated with reduced incidence of AD [60]. Controvertibly, dementia and AD development in older adults has been linked to low plasma leptin levels [61]. Some studies have proved inconclusive regarding a difference in leptin between AD patients and controls, unless an ApoE4 allele was present [62].

Clinical attention to leptin has indicated that there is a statistical trend for increased cognitive scores to be associated with lower plasma leptin levels [58]. Interestingly, when stratified by race and gender, excessive leptin in white men was associated with higher cognitive scores, as assessed by the Montreal Cognitive Assessment, while the opposite trend persisted amongst black men [58]. AD patients with excessive low weight and decreased leptin plasma levels, implicates a hypothalamic level malfunction [63]. Using the Modified Mini Mental State Exam, there is shown to be a protective quality of cellular leptin combating cognitive decline [64], [65].

Finally, the increased gender difference regarding the incidence of AD in women can potentially be linked to the increased relative loss of adipokines in women than in men. Further gender differences in leptin occur regarding C-reactive protein (CRP) levels, highly correlated to leptin levels in women but not in men, which potentially links increased CRP levels to the increased relative adipose load for women [66]. Increased abdominal fat in Japanese men has been associated with mild cognitive impairment, the association inconclusive for Japanese women [67].

We are cognizant of the extensive literature related to the effects of a number of hormones that we studied in this article and their respective roles of influencing leptin function: LH [68], [69]; FSH [70]–[72]; estradiol [61], [63], [72].

## Limitations

Although we started with a cohort of 796, after sub analysis, the lowest N was 194 due to missing values. Therefore, we caution any definitive interpretation of these data. Of the remaining 194 subjects, only 159 subjects had leptin values available for statistical analysis against P300 values. While we are encouraging more research in this area of important investigation, we suspect that leptin resistance may have a negative influence on global cognition in women based on the limited cohort of 159 women that was investigated.

We are cognizant that P300 latency may change as a function of pharmaceutical treatments, anxiety, and other physiological and behavioral factors. Thus, these factors must be considered seriously, and we propose caution in interpreting these data. Subsequently, in future studies we will also include Mini-Mental State Examination status, Wechsler Memory Scale tests, and other neurocognitive measures [73] along with electrophysiological measurements.

In pre- and post-menopausal women, we attempted to determine the relationship between plasma leptin levels and brain functionality as measured by P300. However, a PubMed literature search on 10-27-2013 revealed no articles on P300 measures and plasma leptin levels, in spite of many references associating leptin levels and genes with cognition [74]–[79].

Specific demographics and an investigation of correlated BMI levels with leptin should be further expanded in future studies. We were unable to include additional demographic data such as body weight, height, and BMI among other fields. We are aware that these additional parameters may add to this study and better evaluate the changes in body composition among menopausal women in addition to cognitive function. A specialized study of this nature would be a significant follow-up to our current observations.

## Conclusions

We are proposing that since leptin has been considered to be a cognitive enhancer, whereby cellular leptin acts on hippocampal cells (as one example) and improves performance in object recognition; leptin resistance and subsequent higher plasma levels should induce cognitive decline. As we expected, higher plasma leptin levels significantly and positively associated with a prolonged latency, which is a determinant for cognitive function.

Thus, our findings show significant association of higher plasma leptin levels, potentially due to leptin resistance, and prolonged P300 latency appears to be the first of such an association. Leptin resistance may delay electrophysiological processing of memory and attention.

## References

[1] Mahmud K (2010) Natural hormone therapy for menopause. Gynecological Endocrinology 26: 81–85. 19995152 10.3109/09513590903184134

[2] Diamanti-Kandarakis E, Lambrinoudaki I, Economou F, Christou M, Piperi C, et al (2010) Androgens associated with advanced glycation end-products in postmenapausal women. Menopause 17: 1–5. 10.1097/gme.0b013e3181e170af 20647960

[3] O'Connor KA, Ferrell R, Brindle E, Trumble B, Shofer J, et al (2009) Progesterone and ovulation across stages of the transition to menopause. Menopause 16: 1178–87. 19568209 10.1097/gme.0b013e3181aa192d PMC2783957

[4] Panay N, Al-Azzawi F, Bouchard C, Davis SR, Eden J, et al (2010) Testosterone treatment of HSDD in naturally menopausal women: the ADORE study. Climacteric 13: 121–31. 20166859 10.3109/13697131003675922

[5] Berent-Spillson A, Persad CC, Love T, Tkaczyk A, Wang H, et al (2010) Early menopausal hormone use influences brain regions used for visual working memory. Menopause 17: 692–9. 20300040 10.1097/gme.0b013e3181cc49e9 PMC2901395

[6] Newhouse PA, Dumas J, Wilkins H, Coderre E, Sites CK, et al (2010) Estrogen treatment impairs cognitive performance after psychosocial stress and monoamine depletion in postmenopausal women. Menopause. 17: 860–73. 10.1097/gme.0b013e3181e15df4 PMC2943238 20616673

[7] Lord C, Engert V, Lupien SJ, Pruessner JC (2010) Effect of sex and estrogen therapy on the aging brain: a voxel-based morphometry study. Menopause 17: 846–51. 20616671 10.1097/gme.0b013e3181e06b83

[8] Wiacek M, Hagner W, Zubrzycki IZ (2010) Measures of menopause driven differences in levels of blood lipids, follicle-stimulating hormone, and luteinizing hormone in women aged 35 to 60 years: National Health and Nutrition Examination Survey III and National Health and Nutrition Examination Survey 1999–2002 study. Menopause 18: 1–7. 10.1097/gme.0b013e3181e7060b 20647956

[9] Bryan KJ, Mudd JC, Richardson SL, Chang J, Lee HG, et al (2010) Down-regulation of serum gonadotropins is as effective as estrogen replacement at improving menopause-associated cognitive deficits. J Neurochem 112: 870–81. 19943850 10.1111/j.1471-4159.2009.06502.x PMC2886127

[10] Kåss AS, Lea TE, Torjesen PA, Gulseth HC, Førre ØT (2010) The association of luteinizing hormone and follicle-stimulating hormone with cytokines and markers of disease activity in rheumatoid arthritis: a case-control study. Scand J Rheumatol 39: 109–17. 20337546 10.3109/03009740903270607

[11] Cham M, Chow C, Hamson DK, Lieblich SE, Galea LA (2014) Effects of chronic estradiol, progesterone and medroxyprogesterone acetate on hippocampal neurogenesis and adrenal mass in adult female rats. J Neuroendocrinol 10.1111/jne.12159 24750490

[12] Wira CR, Fahey JV, Rodriguez-Garcia M, Shen Z, Patel MV (2014) Regulation of mucosal immunity in the female reproductive tract: the role of sex hormones in immune protection against sexually transmitted pathogens. Am J Reprod Immunol 10.1111/aji.12252 PMC4351777 24734774

[13] Anderer P, Saletu B, Gruber D, Linzmayer L, Semlitsch HV, et al (2005) Age-related cognitive decline in the menopause: effects of hormone replacement therapy on cognitive event-related potentials. Maturitas 51: 254–69. 15978969 10.1016/j.maturitas.2004.08.005

[14] Gibbs RB (2010) Estrogen therapy and cognition: a review of the cholinergic hypothesis. Endocr Rev 31: 224–53. 20019127 10.1210/er.2009-0036 PMC2852210

[15] Short RA, Bowen RL, O'Brien PC, Graff-Radford NR (2001) Elevated gonadotropin levels in patients with Alzheimer disease. Mayo Clin Proc 76: 906–9. 11560301 10.4065/76.9.906

[16] Al-Hader AA, Tao YX, Lei ZM, Rao CV (1997) Fetal rat brains contain luteinizing hormone/human chorionic gonadotropin receptors. Early Pregnancy 3(4): 323–9. 10086084

[17] Wich BK, Carnes M (1995) Menopause and the aging female reproductive system. Endocrinol Metab Clin North Am 24: 273–95. 7656892

[18] Foster JD, Strauss JF 3rd, Paavola LG (1993) Cellular events involved in hormonal control of receptor-mediated endocytosis: regulation occurs at multiple sites in the low-density lipoprotein pathway, including steps beyond the receptor. Endocrinology 132(1): 337–50. 8419131 10.1210/endo.132.1.8419131

[19] Davis JD, Podolanczuk A, Donahue JE, Stopa E, Hennessey JV, et al (2008) Thyroid hormone levels in the prefrontal cortex of post-mortem brains of Alzheimer's disease patients. Curr Aging Sci 1: 175–81. 20021390 10.2174/1874609810801030175 PMC2953721

[20] El Kholy M, Fahmi ME, Nassar AE, Selim S, Elsedfy HH (2007) Prevalence of minor musculoskeletal anomalies in children with congenital hypothyroidism. Horm Res 68: 272–5. 17587855 10.1159/000104175

[21] Sözay S, Gökçe-Kutsal Y, Celiker R, Erbas T, Başgöze O (1994) Neuroelectrophysiological evaluation of untreated hyperthyroid patients. Thyroidology 6: 55–9. 7536451

[22] Zilberman JM (2011) Association between menopause, obesity, and cognitive impairment. ScienceDaily. Available: http://www.sciencedaily.com/releases/2011/10/111013141824.htm. Accessed 2012 Jul 11.

[23] Priya T, Chowdhury MG, Vasanth K, Vijayakumar TM, Ilango K, et al (2013) Assessment of serum leptin levels in association with the metabolic risk factors of pre- and post- menopausal rural women in South India. Diabetes Metab Syndr 7(4): 233–7. 24290091 10.1016/j.dsx.2013.06.016

[24] Kocyigit H, Bal S, Atay A, Koseoglu M, Gurgan A (2013) Plasma leptin values in postmenopausal women with osteoporosis. Bosn J Basic Med Sci 13(3): 192–6. 23988172 10.17305/bjbms.2013.2361 PMC4333977

[25] Abbenhardt C, McTiernan A, Alfano CM, Wener MH, Campbell KL, et al (2013) Effects of individual and combined dietary weight loss and exercise interventions in postmenopausal women on adiponectin and leptin levels. J Intern Med 274(2): 163–75. 23432360 10.1111/joim.12062 PMC3738194

[26] Iida T, Domoto T, Takigawa A, Nakamura S, Kato Y, et al (2011) Relationships among blood leptin and adiponectin levels, fat mass, and bone mineral density in Japanese pre- and postmenopausal women. Hiroshima J Med Sci 60(4): 71–8. 22389950

[27] Ruszkowska B, Sokup A, Kulwas A, Socha MW, Goralczyk K, et al (2012) Assessment of ghrelin and leptin receptor levels in postmenopausal women who received oral or transdermal menopausal hormonal therapy. J Zhejiang Univ Sci B 13(1): 35–42. 22205618 10.1631/jzus.B1100276 PMC3251750

[28] Lee SW, Jo HH, Kim MR, You YO, Kim JH (2012) Association Between metabolic syndrome and serum leptin levels in postmenopausal women. J Obstet Gynaecol 32(1): 73–7. 22185543 10.3109/01443615.2011.618893

[29] Ben A, Jemaa R, Ftouhi B, Kallel A, Feki M, et al (2011) Relationship of plasma leptin and adiponectin concentrations with menopausal status in Tunisian women. Cytokine 56(2): 338–42. 21802312 10.1016/j.cyto.2011.06.026

[30] Soni AC, Conroy MB, Mackey RH, Kueller LH (2011) Ghrelin, leptin, adiponectin, and insulin levels and concurrent and future weight change in overweight, postmenopausal women. Menopause 18(3): 296–301. 21449093 10.1097/gme.0b013e3181f2e611 PMC3069721

[31] Sarac F, Oztekin K, Celebi G (2011) Early menopause association with employment, smoking, divorced marital status and low leptin levels. Gynecol Endocrinol 27(4): 273–8. 20528208 10.3109/09513590.2010.491165

[32] Marques-Vidal P, Bochud M, Paccaud F, Mooser V, Waeber G, et al (2010) Distribution of plasma levels and adiponectin and leptin in an adult Caucasian population. Clin Endocrinol (Oxf) 72(1): 38–46. 19473178 10.1111/j.1365-2265.2009.03628.x

[33] Nicklas B, Toth MJ, Goldberg AP, Poehlman ET (1997) Racial differences in plasma leptin concentrations in obese postmenopausal women. J Clin Endocrinol Metab 82(1): 315–7. 8989280 10.1210/jcem.82.1.3659

[34] Forbes GB, Welle SL (1983) Lean body mass in obesity. Int J Obes 7(2): 99–107. 6862762

[35] Fischer B, Gleason C, Asthana S (2014) Effects of hormone therapy on cognition and mood. Fertil Steril 101(4): 898–904. 24680649 10.1016/j.fertnstert.2014.02.025 PMC4330961

[36] Eramudugolla R, Bielak AA, Bunce D, Easteal S, Cherbuin N, et al (2014) Long-term cognitive correlates of traumatic brain injury across adulthood and interactions with APOE genotype, sex and age cohorts. J Int Neuropsychol Soc 20(4): 444–454. 24670469 10.1017/S1355617714000174

[37] Li R, Cui J, Shen Y (2014) Brain sex matters: estrogen in cognition and Alzheimer's disease. Mol Cell Endocrinol 10.1016/j.mce.2013.12.018 PMC4040318 24418360

[38] Shah NR, Braverman ER (2012) Measuring adiposity in patients: the utility of body mass index (BMI), percent body fat, and leptin. PLoS One 7(4): e33308. 22485140 10.1371/journal.pone.0033308 PMC3317663

[39] Arnoldussen IA, Kiliaan AJ, Gustafson DR (2014) Obesity and dementia: adipokines interact with the brain. Eur Neuropsychopharmacol 10.1016/j.euroneuro.2014.03.002 PMC4169761 24704273

[40] Bove RM, Brick DJ, Healy BC, Mancuso SM, Gerweck AV, et al (2013) Metabolic and endocrine correlates of cognitive function in healthy young women. Obesity (Silver Spring) 21(7): 1343–1349. 23671055 10.1002/oby.20212 PMC3742554

[41] Gunstad J, Spitznagel MB, Keary TA, Glickman E, Alexander T, et al (2008) Serum leptin levels are associated with cognitive function in older adults. Brain Res 1230: 233–236. 18675793 10.1016/j.brainres.2008.07.045

[42] Zhou X, Chai Y, Chen K, Yang Y, Liu Z (2013) A meta-analysis of reference values of leptin concentration in healthy postmenopausal women. PLoS One 8(8): e72734. 24023638 10.1371/journal.pone.0072734 PMC3758328

[43] Kocyigit H, Bal S, Atay A, Koseoglu M, Gurgan A (2013) Plasma leptin values in postmenopausal women with osteoporosis. Bosn J Basic Med Sci 13(3): 192–196. 23988172 10.17305/bjbms.2013.2361 PMC4333977

[44] Thurston RC, Chang Y, Mancuso P, Matthews KA (2013) Adipokines, adiposity, and vasomotor symptoms during the menopause transition: findings from the Study of Women's Health Across the Nation. Fertil Steril 100(3): 793–800. 23755948 10.1016/j.fertnstert.2013.05.005 PMC3759568

[45] Jones ME, Schoemaker M, Rae M, Folkard EJ, Dowsett M, et al (2013) Changes in estradiol and testosterone levels in postmenopausal women after changes in body mass index. J Clin Endocrinol Metab 98(7): 2967–2974. 23666973 10.1210/jc.2013-1588

[46] Whitmer RA, Gunderson EP, Barrett-Connor E, Quesenberry CP, Yaffe K (2005) Obesity in middle age and future risk of dementia: A 27 year longitudinal population based study. BMJ 330(7504): 1360. 15863436 10.1136/bmj.38446.466238.E0 PMC558283

[47] Thurston RC, Chang Y, Mancuso P, Matthews KA (2013) Adipokines, adiposity, and vasomotor symptoms during the menopause transition: findings from the Study of Women's Health Across the Nation. Fertil Steril 100(3): 793–800. 23755948 10.1016/j.fertnstert.2013.05.005 PMC3759568

[48] Beydoun MA, Beydoun HA, Wang Y (2008) Obesity and central obesity as risk factors for incident dementia and its subtypes: A systematic review and meta-analysis. Obes Rev 9(3): 204–18. 18331422 10.1111/j.1467-789X.2008.00473.x PMC4887143

[49] Hassing LB, Dahl AK, Pedersen NL, Johansson B (2010) Overweight in midlife is related to lower cognitive function 30 years later: A prospective study with longitudinal assessments. Dement Geriatr Cogn Disord 29(6): 543–52. 20606436 10.1159/000314874 PMC3202952

[50] Buchman AS, Wilson RS, Bienias JL, Shah RC, Evans DA, et al (2005) Change in body mass index and risk of incident alzheimer disease. Neurology 65(6): 892–7. 16186530 10.1212/01.wnl.0000176061.33817.90

[51] Cronk BB, Johnson DK, Burns JM (2010) Body mass index and cognitive decline in mild cognitive impairment. Alzheimer Dis Assoc Disord 24(2): 126–30. 19571736 10.1097/WAD.0b013e3181a6bf3f PMC3068614

[52] Razay G, Vreugdenhil A, Wilcock G (2006) Obesity, abdominal obesity and alzheimer disease. Dement Geriatr Cogn Disord 22: 173–176. 16847377 10.1159/000094586

[53] Friedman JM, Halaas JL (1998) Leptin and the regulation of body weight in mammals. Nature 395: 763–770 10.1038/27376 9796811

[54] Harvey J, Solovyova N, Irving A (2006) Leptin and its role in hippocampal synaptic plasticity. Prog Lipid Res 45: 369–378. 16678906 10.1016/j.plipres.2006.03.001 PMC1762032

[55] Perez-Gonzalez R, Antequera D, Vargas T, Spuch C, Bolos M, et al.. (2011) Leptin induces proliferation of neuronal progenitors and neuroprotection in a mouse model of Alzheimer's disease. J Alzheimers Dis (Suppl 2): 17–25. 10.3233/JAD-2011-102070 21335656

[56] Fewlass DC, Noboa K, Pi-Sunyer FX, Johnston JM, Yan SD, et al (2004) Obesity-related leptin regulates alzheimer's abeta. FASEB J 18: 1870–1878. 15576490 10.1096/fj.04-2572com

[57] Greco SJ, Bryan KJ, Sarkar S, Zhu X, Smith MA, et al (2010) Leptin reduces pathology and improves memory in a transgenic mouse model of Alzheimer's disease. J Alzheimers Di 19: 1155–1167. 10.3233/JAD-2010-1308 PMC2862270 20308782

[58] Yan BC, Choi JH, Yoo KY, Lee CH, Hwang IK, et al (2011) Leptin's neuroprotective action in experimental transient ischemic damage of the gerbil hippocampus is linked to altered leptin receptor immunoreactivity. J Neurol Sci 303: 100–108. 21277586 10.1016/j.jns.2010.12.025

[59] Farr SA, Banks WA, Morley JE (2006) Effects of leptin on memory processing. Peptides 27: 1420–1425. 16293343 10.1016/j.peptides.2005.10.006

[60] Warren MW, Hynan LS, Weiner MF (2012) Leptin and cognition. Dement Geriatr Cogn Disord 33: 410–415. 22814193 10.1159/000339956 PMC3732374

[61] Kanoski SE, Hayes MR, Greenwald HS, Fortin SM, Gianessi CA, et al (2011) Hippocampal leptin signaling reduces food intake and modulates food-related memory processing. Neuropsychopharmacology 36: 1859–1870. 21544068 10.1038/npp.2011.70 PMC3154104

[62] Lieb W, Beiser AS, Vasan RS, Tan ZS, Au R, et al (2009) Association of plasma leptin levels with incident alzheimer disease and MRI measures of brain aging. JAMA 302: 2565–2572. 20009056 10.1001/jama.2009.1836 PMC2838501

[63] Warren MW, Hynan LS, Weiner MF (2012) Lipids and adipokines as risk factors for Alzheimer's disease. J Alzheimers Dis 29: 151–157. 22232009 10.3233/JAD-2012-111385 PMC3732377

[64] Power DA, Noel J, Collins R, O'Neill D (2001) Circulating leptin levels and weight loss in Alzheimer's disease patients. Dement Geriatr Cogn Disord 12: 167–170. 11173891 10.1159/000051252

[65] Holden KF, Lindquist K, Tylavsky FA, Rosano C, Harris TB, et al (2009) Serum leptin level and cognition in the elderly: Findings from the health abc study. Neurobiol Aging 30: 1483–1489. 18358569 10.1016/j.neurobiolaging.2007.11.024 PMC5278645

[66] Kamogawa K, Kohara K, Tabara Y, Uetani E, Nagai T, et al (2010) Abdominal fat, adipose-derived hormones and mild cognitive impairment: The j-shipp study. Dement Geriatr Cogn Disord 30: 432–439. 21088422 10.1159/000321985

[67] Teng EL, Chui HC (1987) The modified mini-mental state (3 ms) examination. J Clin Psychiatry 48: 314–318. 3611032

[68] Abdullah SM, Khera A, Leonard D, Das SR, Canham RM, et al (2007) Sex differences in the association between leptin and CRP: Results from the Dallas heart study. Atherosclerosis 195: 404–410. 17141244 10.1016/j.atherosclerosis.2006.10.022

[69] Victor RG, Haley RW, Willett DL, Peshock RM, Vaeth PC, et al (2004) The Dallas Heart Study: A population-based probability sample for the multidisciplinary study of ethnic differences in cardiovascular health. Am J Cardiol 93: 1473–1480. 15194016 10.1016/j.amjcard.2004.02.058

[70] Chakrabarti J (2013) Serum leptin level in women with polycystic ovary syndrome: correlation with adiposity, insulin, and circulating testosterone. Ann Med Health Sci Res 3: 191–196. 23919188 10.4103/2141-9248.113660 PMC3728861

[71] Siawrys G, Smolinska N (2013) In vitro effects of luteinizing hormone, progesterone and oestradiol-17β on leptin gene expression and leptin secretion by porcine luteal cells obtained in early pregnancy. J Physiol Pharmacol 64: 513–520. 24101399

[72] Geber S, Brandão AH, Sampaio M (2012) Effects of estradiol and FSH on leptin levels in women with suppressed pituitary. Reprod Biol Endocrinol 10: 45–49. 22703959 10.1186/1477-7827-10-45 PMC3495667

[73] Ajala OM, Ogunro PS, Elusanmi GF, Ogunyemi OE, Bolarinde AA (2013) Changes in serum leptin during phases of menstrual cycle of fertile women: relationship to age groups and fertility. Int J Endocrinol Metab 11: 27–33. 23853617 10.5812/ijem.6872 PMC3693651

[74] Fungfuang W, Terada M, Komatsu N, Moon C, Saito TR (2013) Effects of estrogen on food intake, serum leptin levels and leptin mRNA expression in adipose tissue of female rats. Lab Anim Res 29: 168–173. 24106512 10.5625/lar.2013.29.3.168 PMC3791351

[75] Braverman E, Blum K, Damle U, Kerner M, Dushaj K, et al (2013) Evoked potentials and neuropsychological tests validate positron emission topography (PET) in brain metabolism in cognitively impaired patients. PLOS One 8(3): e55398. 23526928 10.1371/journal.pone.0055398 PMC3604004

[76] Nasreddine ZS, Phillips NA, Bedirian V, Charbonneau S, Whitehead V, et al (2005) The Montreal Cognitive Assessment, MOCHA: A brief screening tool for mild cognitive impairment. J Am Geriatr Soc 53: 695–699. 15817019 10.1111/j.1532-5415.2005.53221.x

[77] Ruhl CE, Everhart JE, Ding J, Goodpaster BH, Kanaya AM, et al (2004) Serum leptin concentrations and body adipose measures in older black and white adults. Am J Clin Nutr 80: 576–583. 15321795 10.1093/ajcn/80.3.576

[78] Rance KA, Johnstone AM, Murison S, Duncan JS, Wood SG, et al (2007) Plasma leptin levels are related to body composition, sex, insulin levels and the a55v polymorphism of the ucp2 gene. Int J Obes (Lond) 31: 1311–1318. 17342078 10.1038/sj.ijo.0803535

[79] Kanaya AM, Lindquist K, Harris TB, Launer L, Rosano C, et al (2009) Total and regional adiposity and cognitive change in older adults: The health, aging and body composition (abc) study. Arch Neurol 66: 329–335. 19273751 10.1001/archneurol.2008.570 PMC2739693

[80] Weiner MF (2008) Perspective on race and ethnicity in Alzheimer's disease research. Alzheimers Dement 4: 233–238. 18631972 10.1016/j.jalz.2007.10.016 PMC2570194

[81] Parra M, Lorena Ascencio LL, Urquina HF, Manes F, Ibanez AM (2012) P300 and neuropsychological assessment in mild cognitive impairment and Alzheimer dementia. Front Neur 3: 172. 10.3389/fneur.2012.00172 PMC3514532 23227021

[82] Howe AS, Bani-Fatemi A, De Luca V (2014) The clinical utility of the auditory P300 latency subcomponent event-related potential in preclinical diagnosis of patients with mild cognitive impairment and Alzheimer's disease. Brain Cog 86: 64–74. 10.1016/j.bandc.2014.01.015 24565814

[83] Asaumi Y, Morita K, Nakashima Y, Muraoka A, Uchimura N (2014) Evaluation of P300 components for emotion-loaded visual event-related potential in elderly subjects, including those with dementia. Psychiatry Clin Neurosci 68(7): 558–67. 24447302 10.1111/pcn.12162

[84] Polich J, Herbst KL (2000) P300 as a clinical assay: rationale, evaluation, and findings. Inter Jour Psychophysiology 38(1): 3–19. 10.1016/s0167-8760(00)00127-6 11027791

[85] Lee MS, Lee SH, Moon EO, Moon YJ, Kim S, et al (2013) Neuropsychological correlates of the P300 patients with Alzheimer's disease. Prog Neuropsychopharmacol Biol Psychiatry 40: 62–9. 22940475 10.1016/j.pnpbp.2012.08.009

[86] Zhou X, Chai Y, Chen K, Yang Y, Liu Z (2013) A meta-analysis of reference values of leptin concentration in healthy menopausal women. PLoS One 8(8): e72734. 24023638 10.1371/journal.pone.0072734 PMC3758328

[87] Chen XW, Shi JW, Yang PS, Wu ZQ (2014) Preoperative plasma leptin levels predict delirium in elderly patients after hip fracture surgery. Peptides 57: 31–5. 24787655 10.1016/j.peptides.2014.04.016

